# Supplementary figures and images for: The RNA helicase DDX39B activates FOXP3 RNA splicing to control T regulatory cell fate
Source: eLife. 2023 Jun 1;12:e76927. doi: 10.7554/eLife.76927 (PMC10234631; doi:10.7554/eLife.76927)

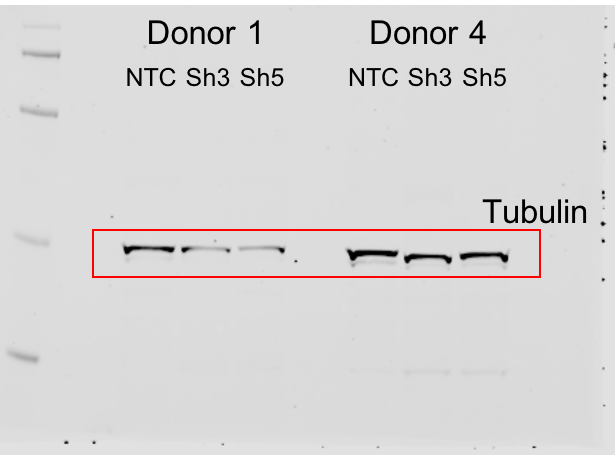

Supplement: Figure 1—figure supplement 5—source data 1. [file elife-76927-fig1-figsupp5-data1.zip › Fig 1_figure supplement 5_associated source files/Fig 1_figure supplement 5_Source Data 3_Labeled.png]

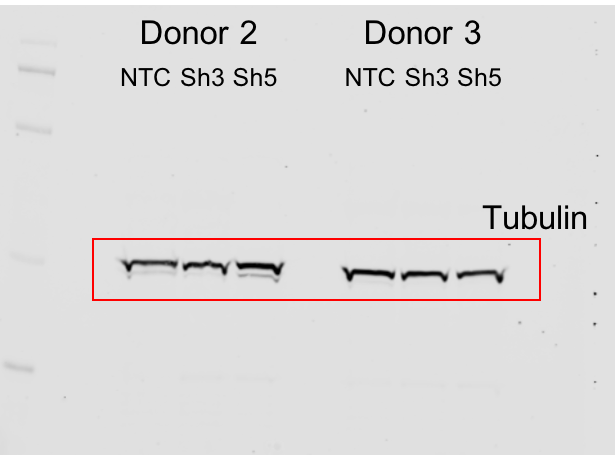

Supplement: Figure 1—figure supplement 5—source data 1. [file elife-76927-fig1-figsupp5-data1.zip › Fig 1_figure supplement 5_associated source files/Fig 1_figure supplement 5_Source Data 6_Labeled.png]

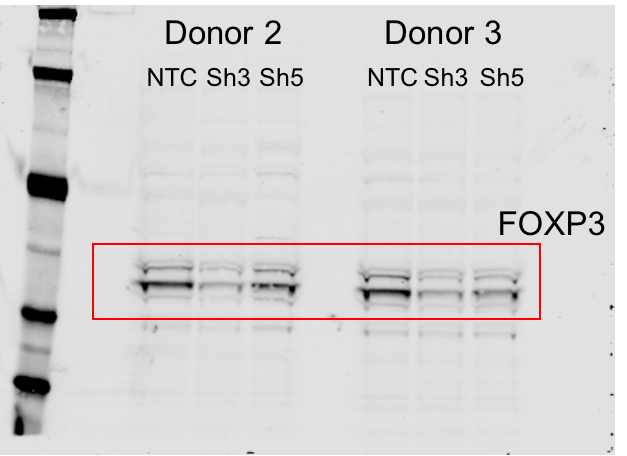

Supplement: Figure 1—figure supplement 5—source data 1. [file elife-76927-fig1-figsupp5-data1.zip › Fig 1_figure supplement 5_associated source files/Fig 1_figure supplement 5_Source Data 5_Labeled.png]

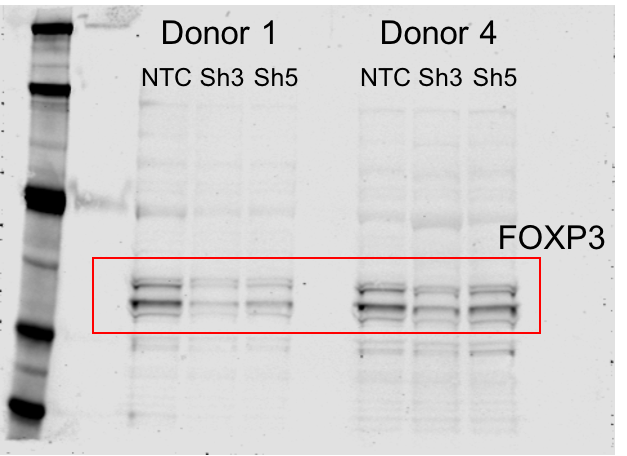

Supplement: Figure 1—figure supplement 5—source data 1. [file elife-76927-fig1-figsupp5-data1.zip › Fig 1_figure supplement 5_associated source files/Fig 1_figure supplement 5_Source Data 2_Labeled.png]

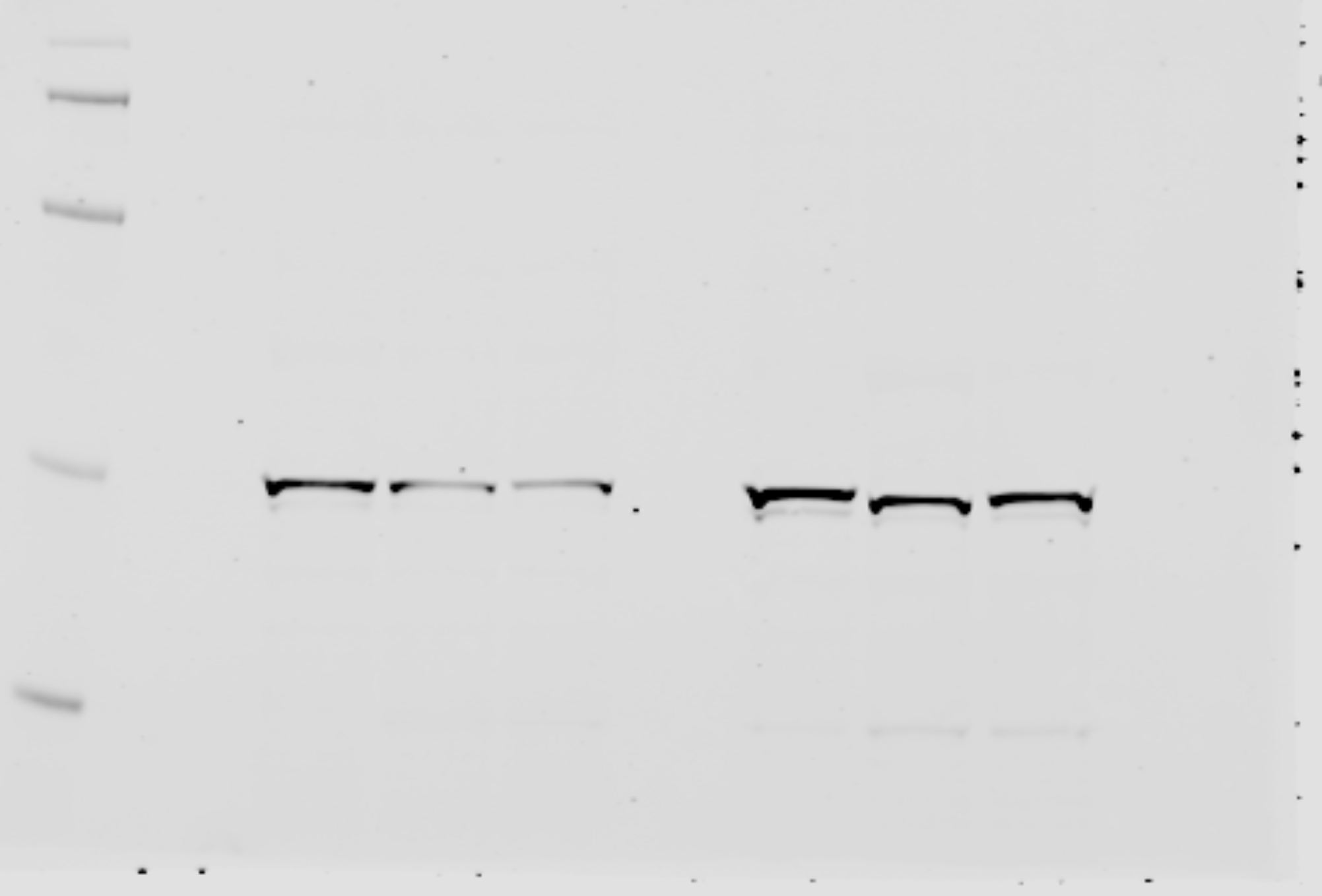

Supplement: Figure 1—figure supplement 5—source data 1. [file elife-76927-fig1-figsupp5-data1.zip › Fig 1_figure supplement 5_associated source files/Fig 1_figure supplement 5_Source Data 3.tif]

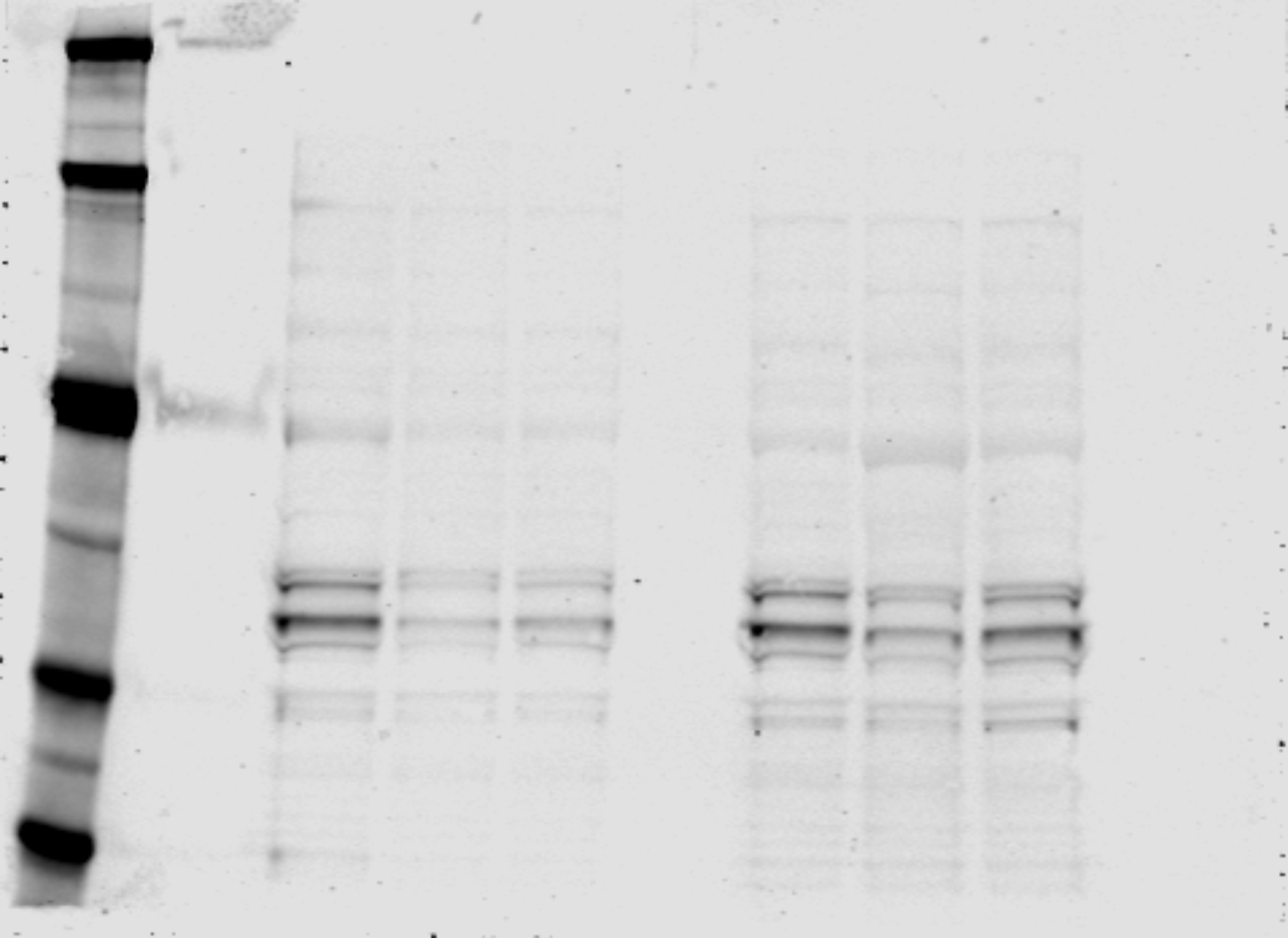

Supplement: Figure 1—figure supplement 5—source data 1. [file elife-76927-fig1-figsupp5-data1.zip › Fig 1_figure supplement 5_associated source files/Fig 1_figure supplement 5_Source Data 2.tif]

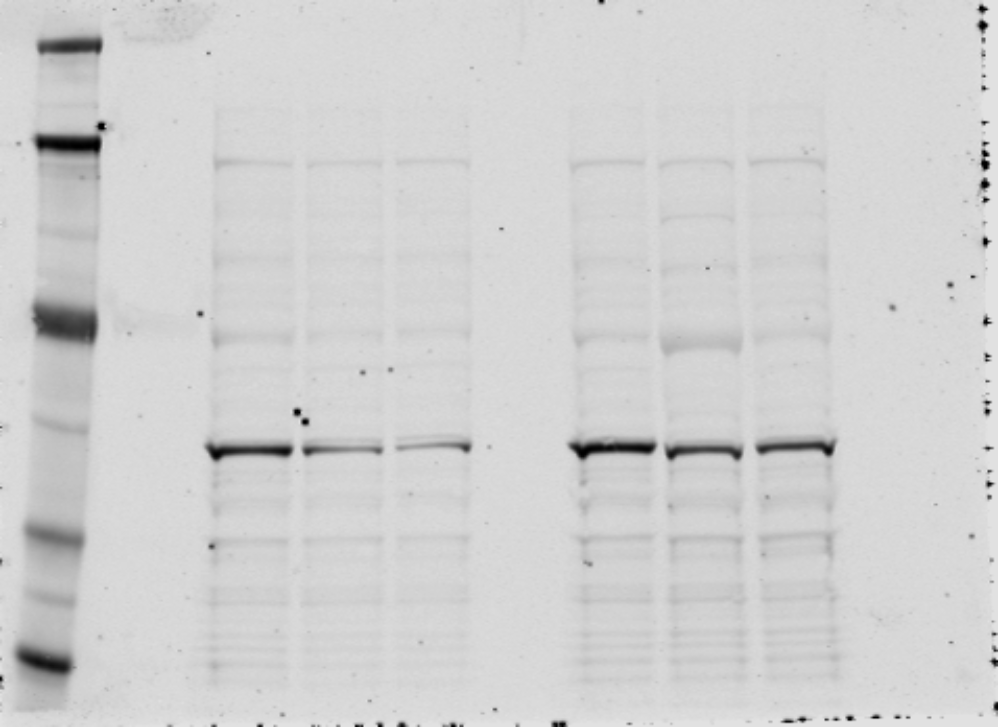

Supplement: Figure 1—figure supplement 5—source data 1. [file elife-76927-fig1-figsupp5-data1.zip › Fig 1_figure supplement 5_associated source files/Fig 1_figure supplement 5_Source Data 1.tif]

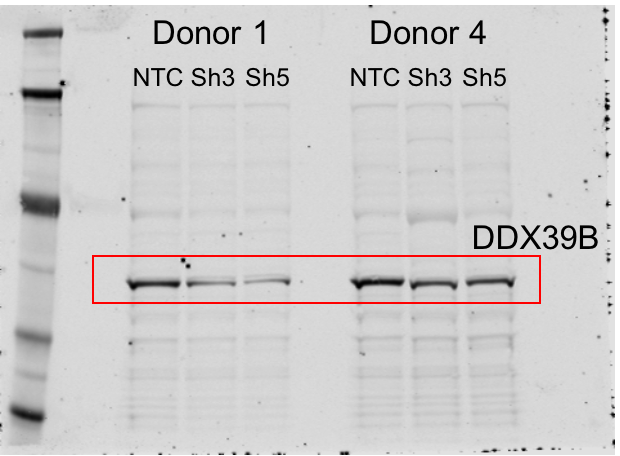

Supplement: Figure 1—figure supplement 5—source data 1. [file elife-76927-fig1-figsupp5-data1.zip › Fig 1_figure supplement 5_associated source files/Fig 1_figure supplement 5_Source Data 1_Labeled.png]

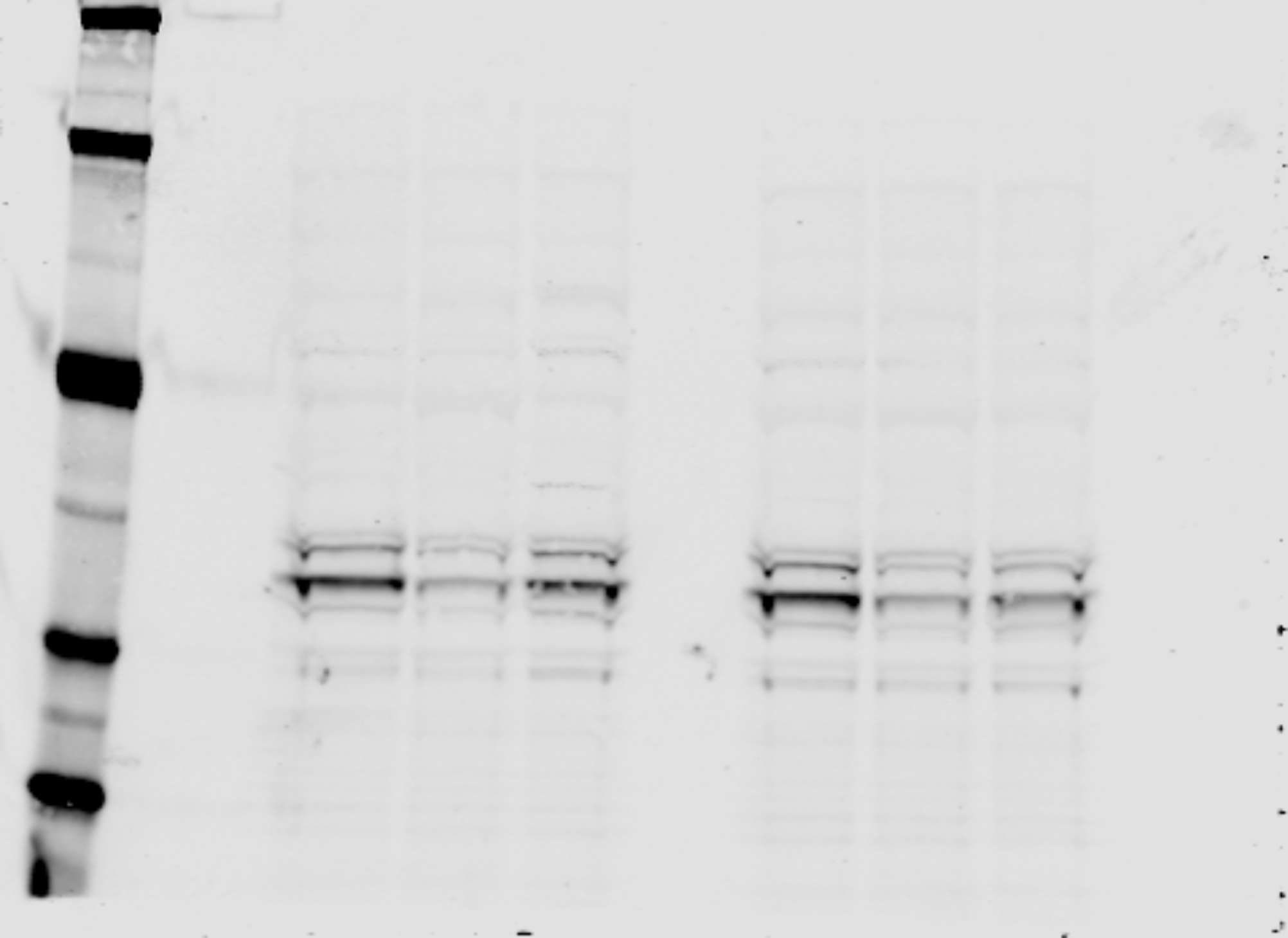

Supplement: Figure 1—figure supplement 5—source data 1. [file elife-76927-fig1-figsupp5-data1.zip › Fig 1_figure supplement 5_associated source files/Fig 1_figure supplement 5_Source Data 5.tif]

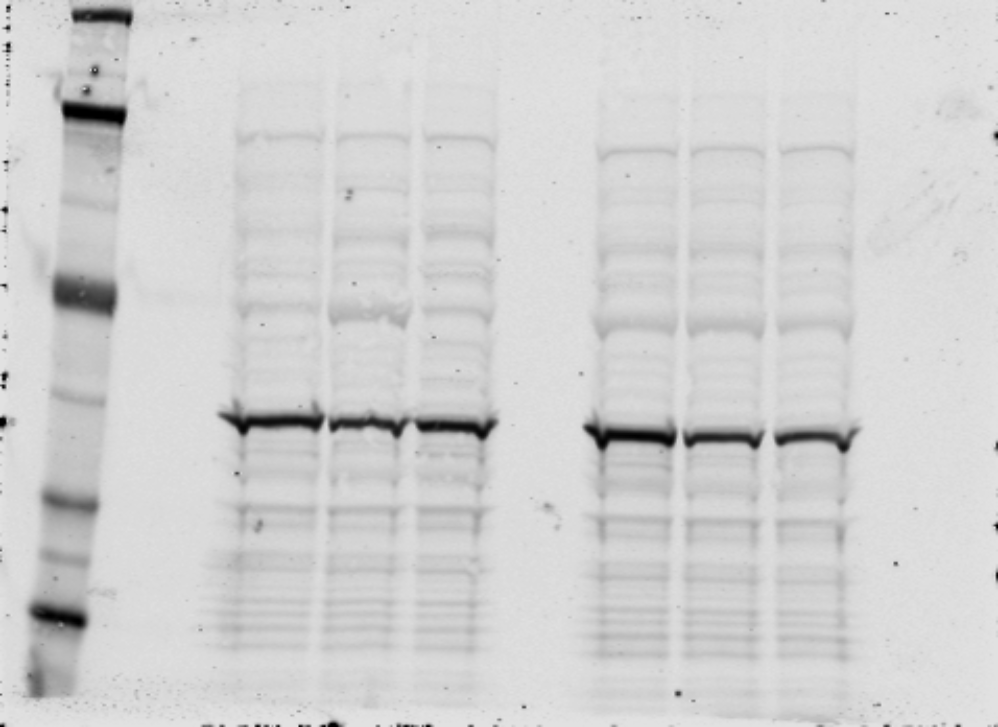

Supplement: Figure 1—figure supplement 5—source data 1. [file elife-76927-fig1-figsupp5-data1.zip › Fig 1_figure supplement 5_associated source files/Fig 1_figure supplement 5_Source Data 4.tif]

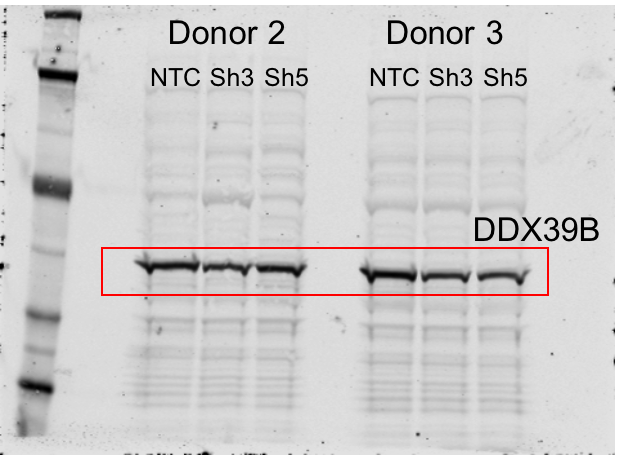

Supplement: Figure 1—figure supplement 5—source data 1. [file elife-76927-fig1-figsupp5-data1.zip › Fig 1_figure supplement 5_associated source files/Fig 1_figure supplement 5_Source Data 4_Labeled.png]

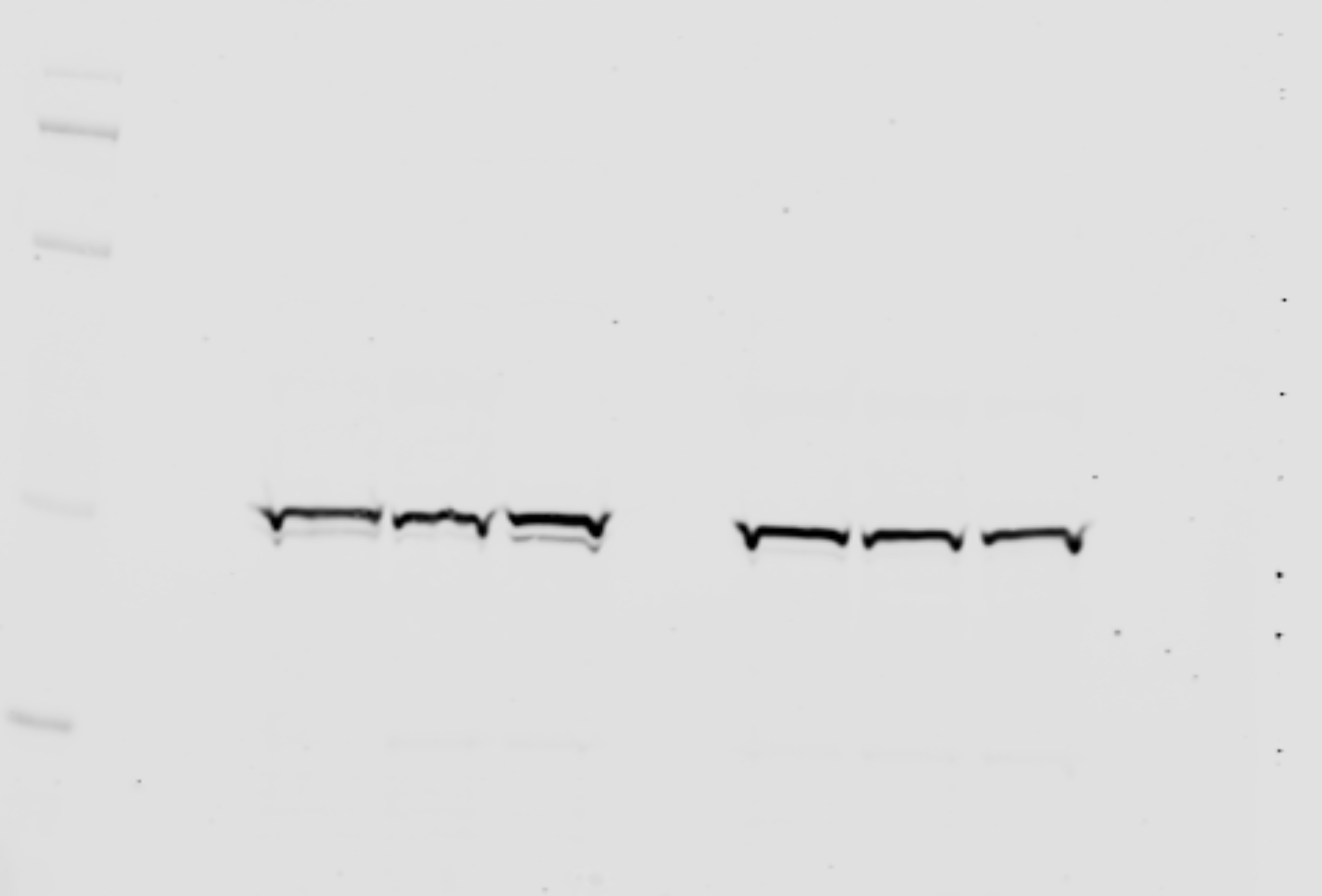

Supplement: Figure 1—figure supplement 5—source data 1. [file elife-76927-fig1-figsupp5-data1.zip › Fig 1_figure supplement 5_associated source files/Fig 1_figure supplement 5_Source Data 6.tif]

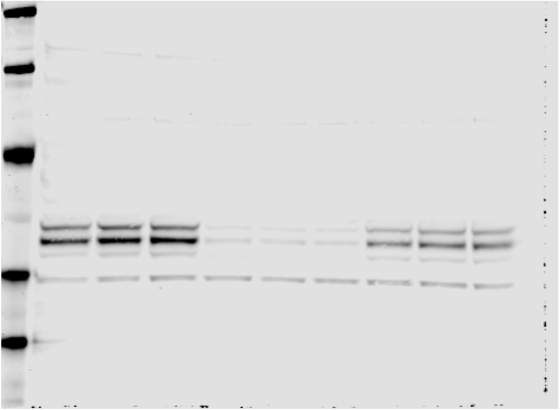

Supplement: Figure 2—source data 1. [file elife-76927-fig2-data1.zip › Fig 2_associated source files/Fig 2C_Source Data 2.png]

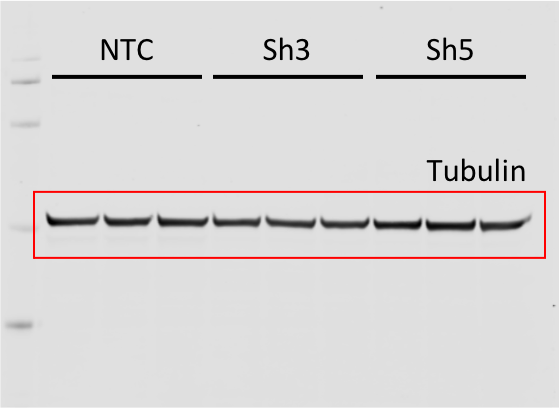

Supplement: Figure 2—source data 1. [file elife-76927-fig2-data1.zip › Fig 2_associated source files/Fig 2C_Source Data 3_Labeled.png]

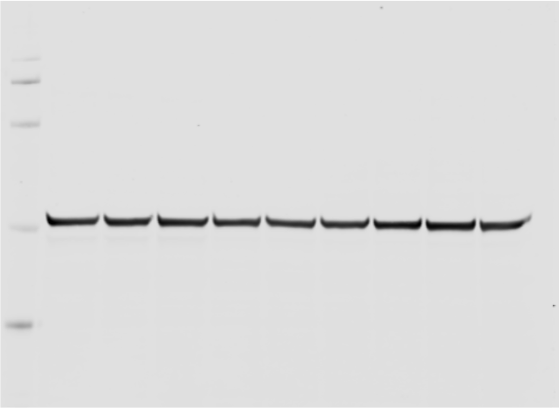

Supplement: Figure 2—source data 1. [file elife-76927-fig2-data1.zip › Fig 2_associated source files/Fig 2C_Source Data 3.png]

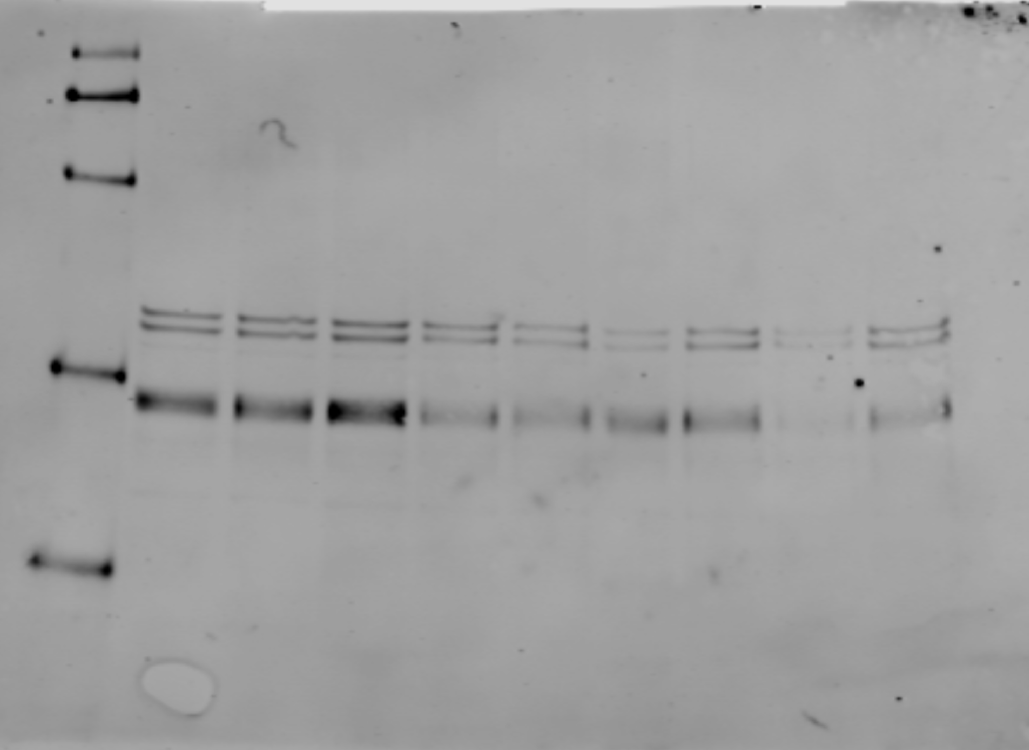

Supplement: Figure 2—source data 1. [file elife-76927-fig2-data1.zip › Fig 2_associated source files/Fig 2E_Source Data 1.tif]

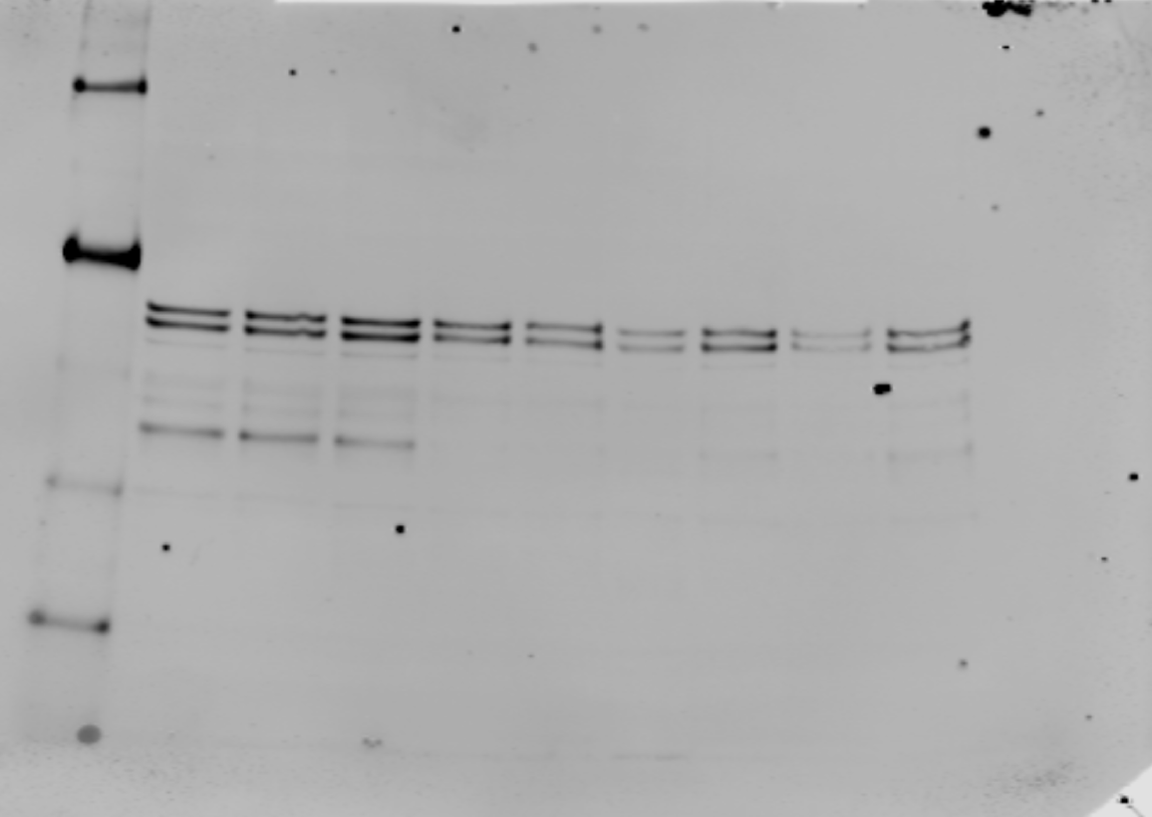

Supplement: Figure 2—source data 1. [file elife-76927-fig2-data1.zip › Fig 2_associated source files/Fig 2E_Source Data 3.tif]

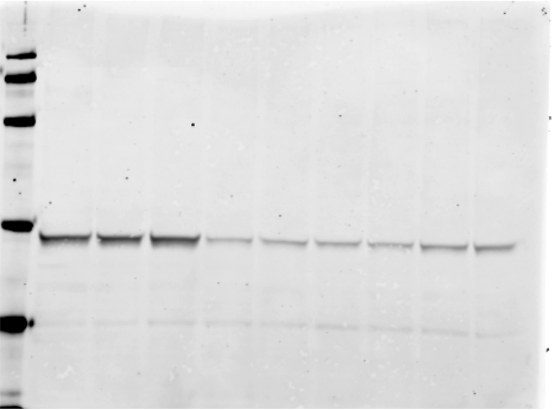

Supplement: Figure 2—source data 1. [file elife-76927-fig2-data1.zip › Fig 2_associated source files/Fig 2C_Source Data 1.png]

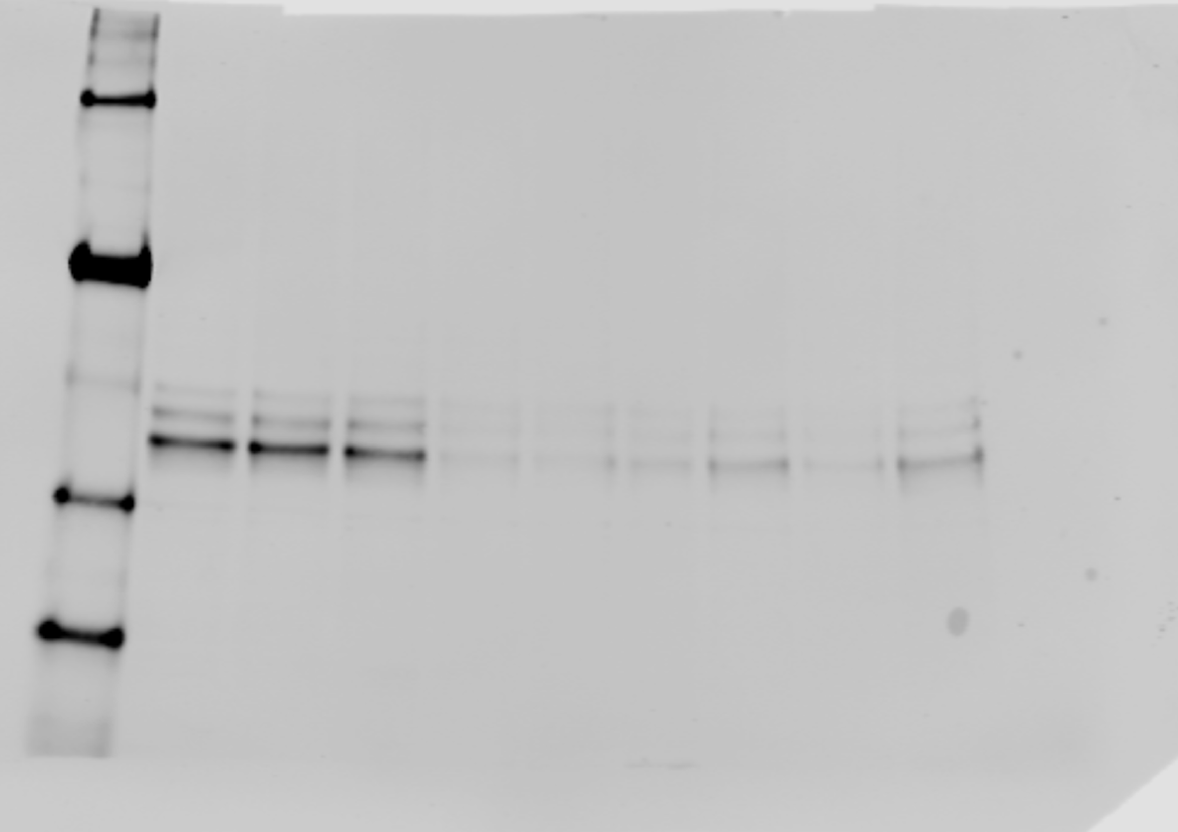

Supplement: Figure 2—source data 1. [file elife-76927-fig2-data1.zip › Fig 2_associated source files/Fig 2E_Source Data 2.tif]

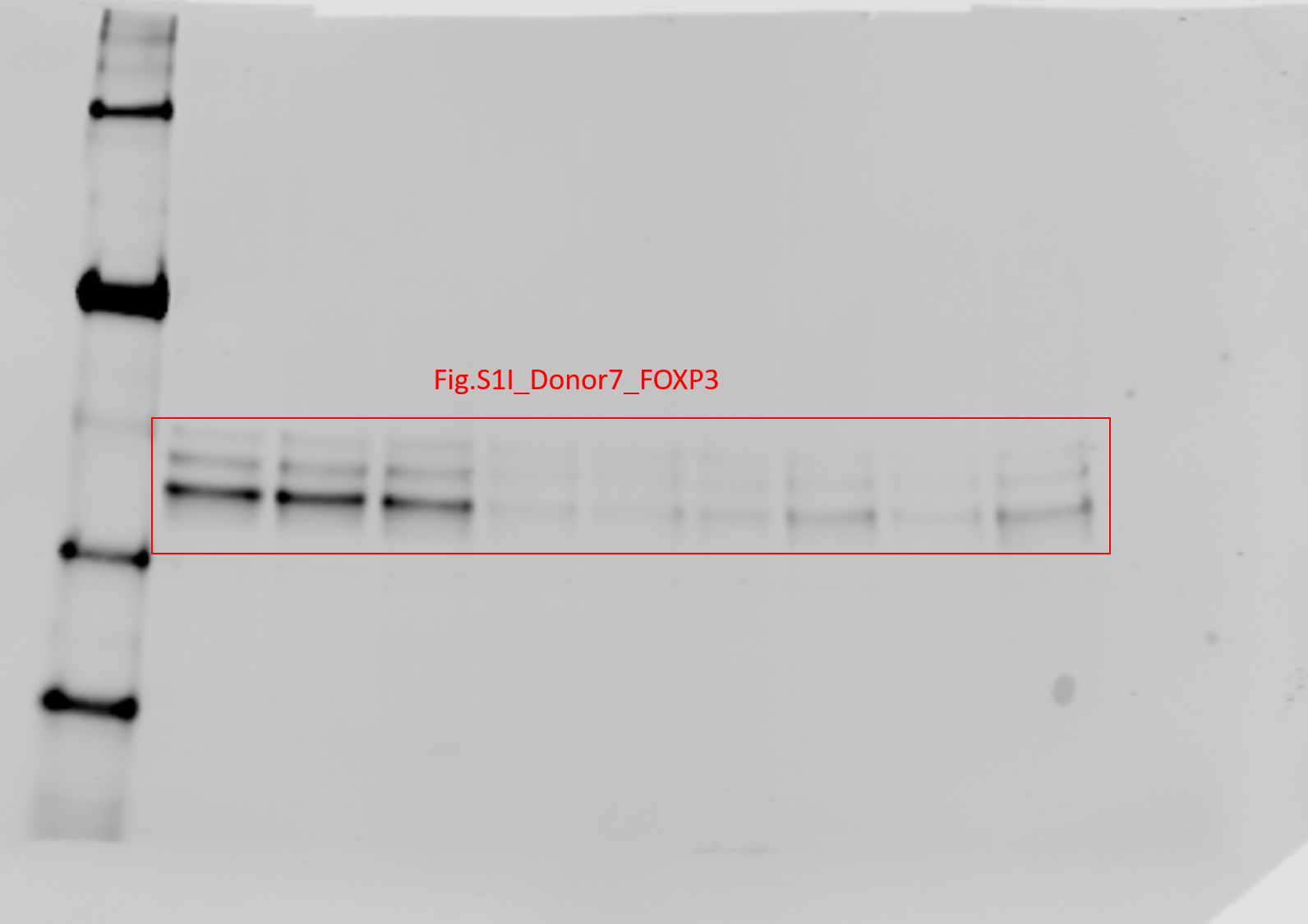

Supplement: Figure 2—source data 1. [file elife-76927-fig2-data1.zip › Fig 2_associated source files/Fig 2E_Source Data 2_Labeled.tif]

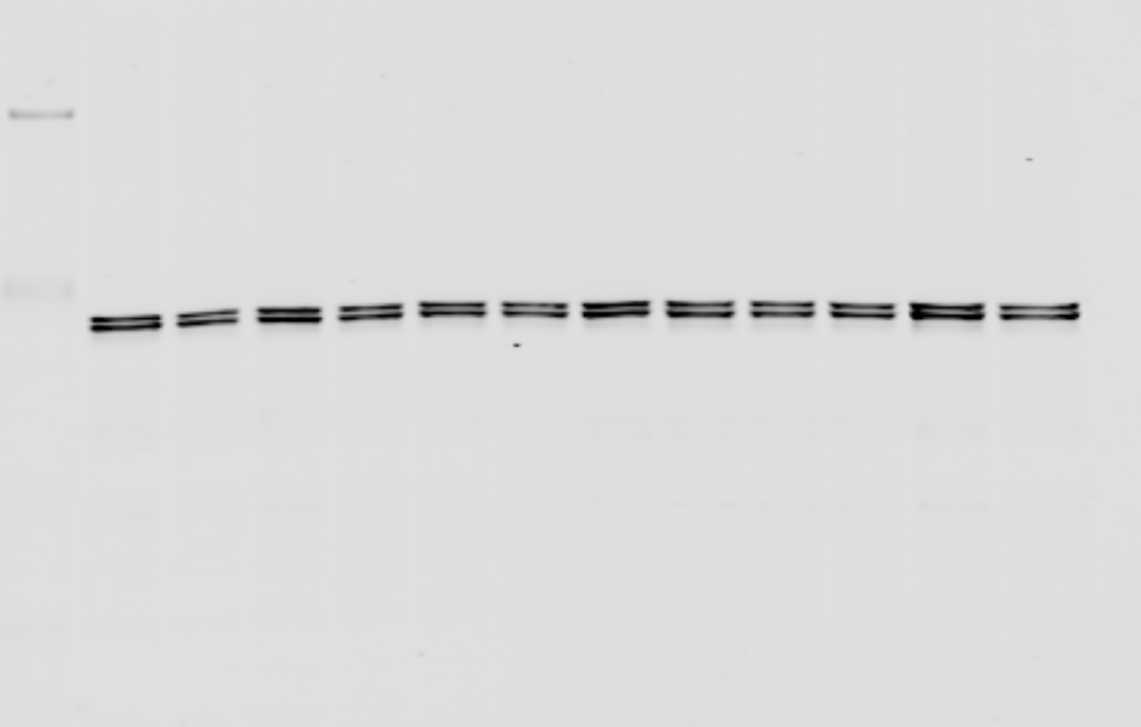

Supplement: Figure 2—source data 1. [file elife-76927-fig2-data1.zip › Fig 2_associated source files/Fig 2E_Source Data 6.tif]

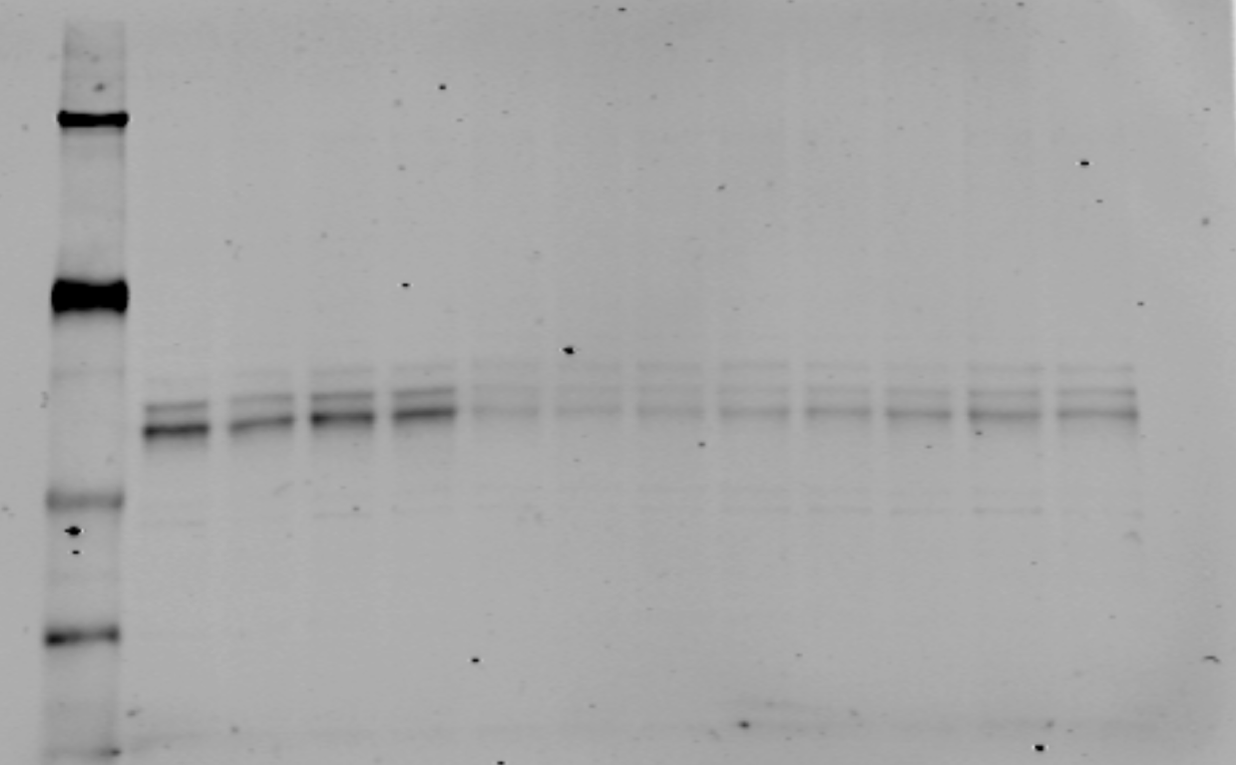

Supplement: Figure 2—source data 1. [file elife-76927-fig2-data1.zip › Fig 2_associated source files/Fig 2E_Source Data 5.tif]

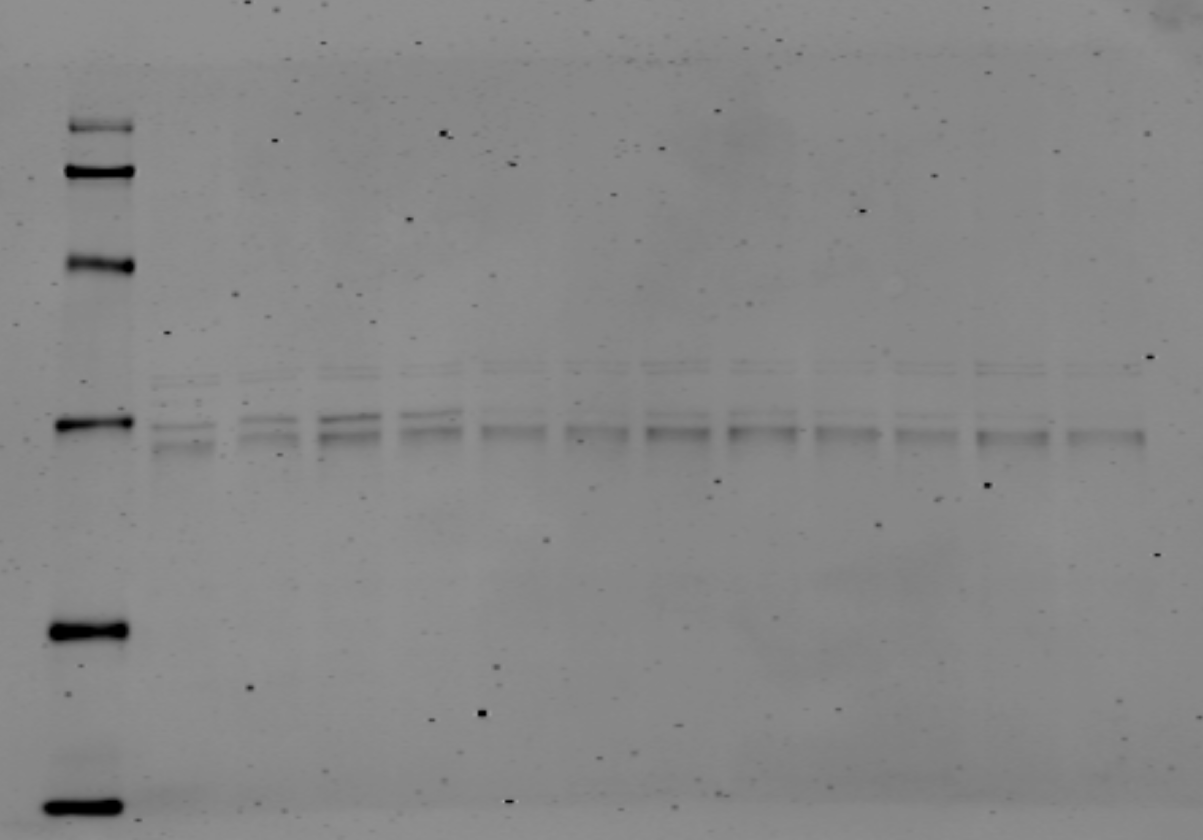

Supplement: Figure 2—source data 1. [file elife-76927-fig2-data1.zip › Fig 2_associated source files/Fig 2E_Source Data 4.tif]

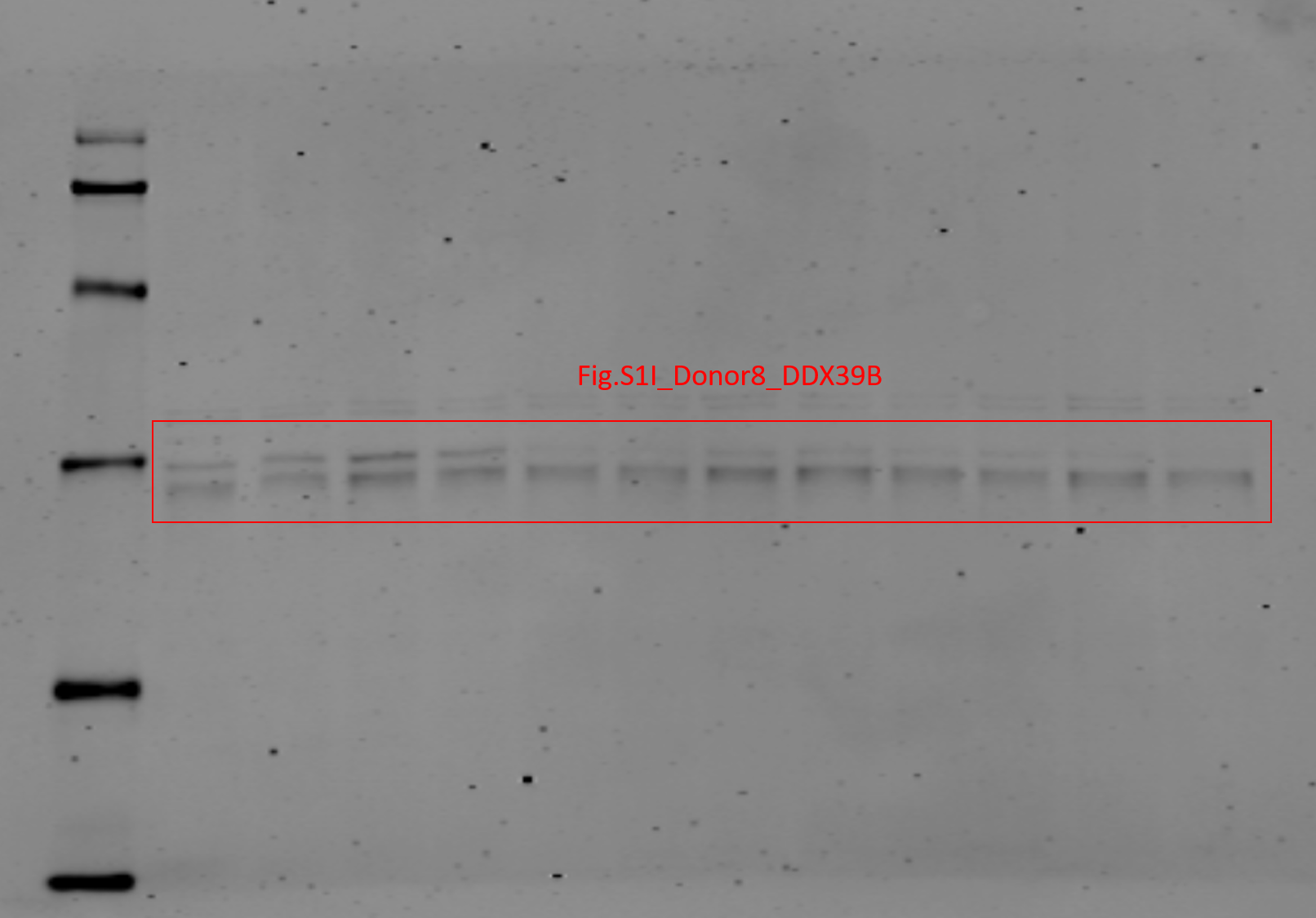

Supplement: Figure 2—source data 1. [file elife-76927-fig2-data1.zip › Fig 2_associated source files/Fig 2E_Source Data 4_Labeled.tif]

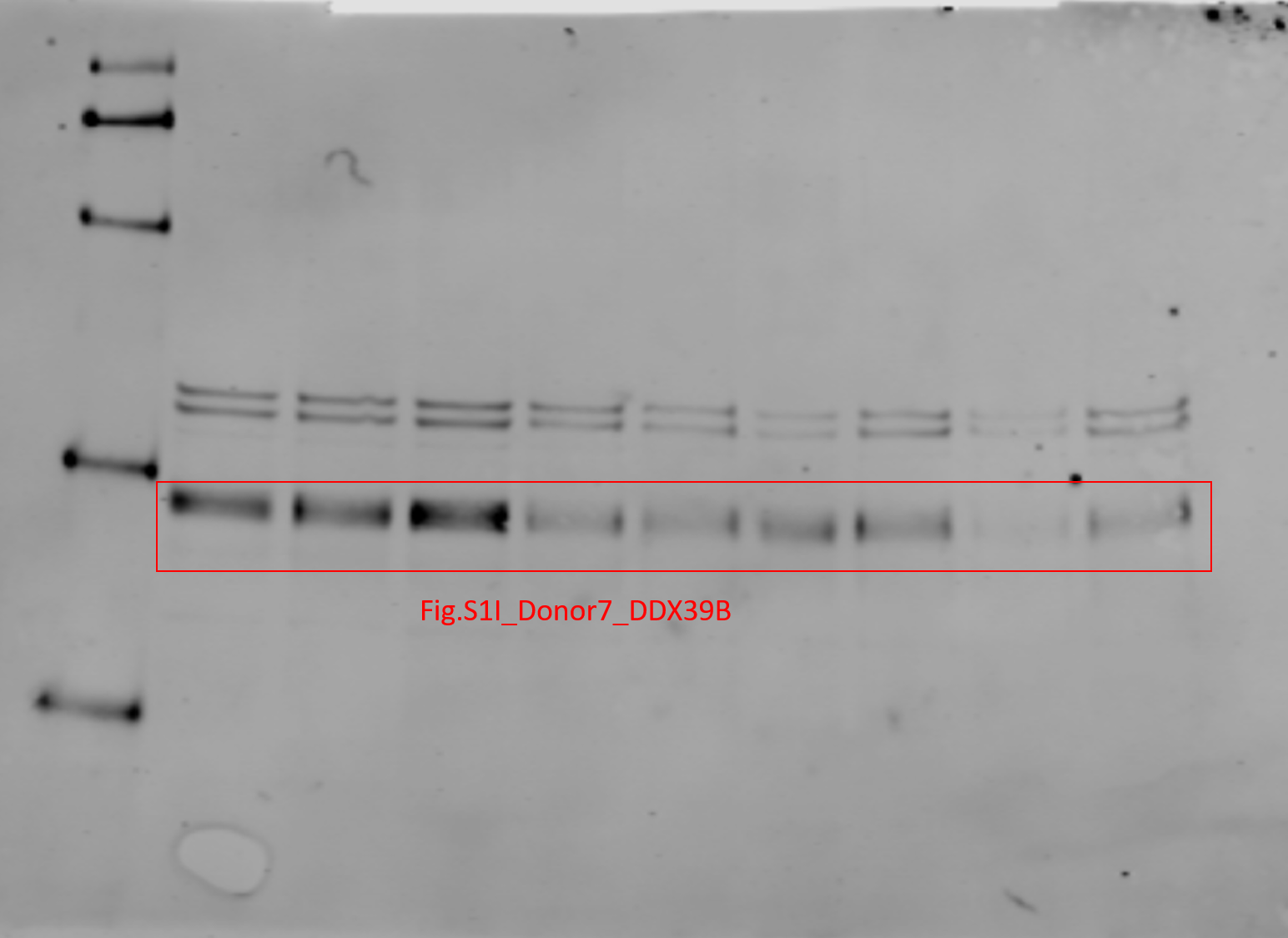

Supplement: Figure 2—source data 1. [file elife-76927-fig2-data1.zip › Fig 2_associated source files/Fig 2E_Source Data 1_Labeled.tif]

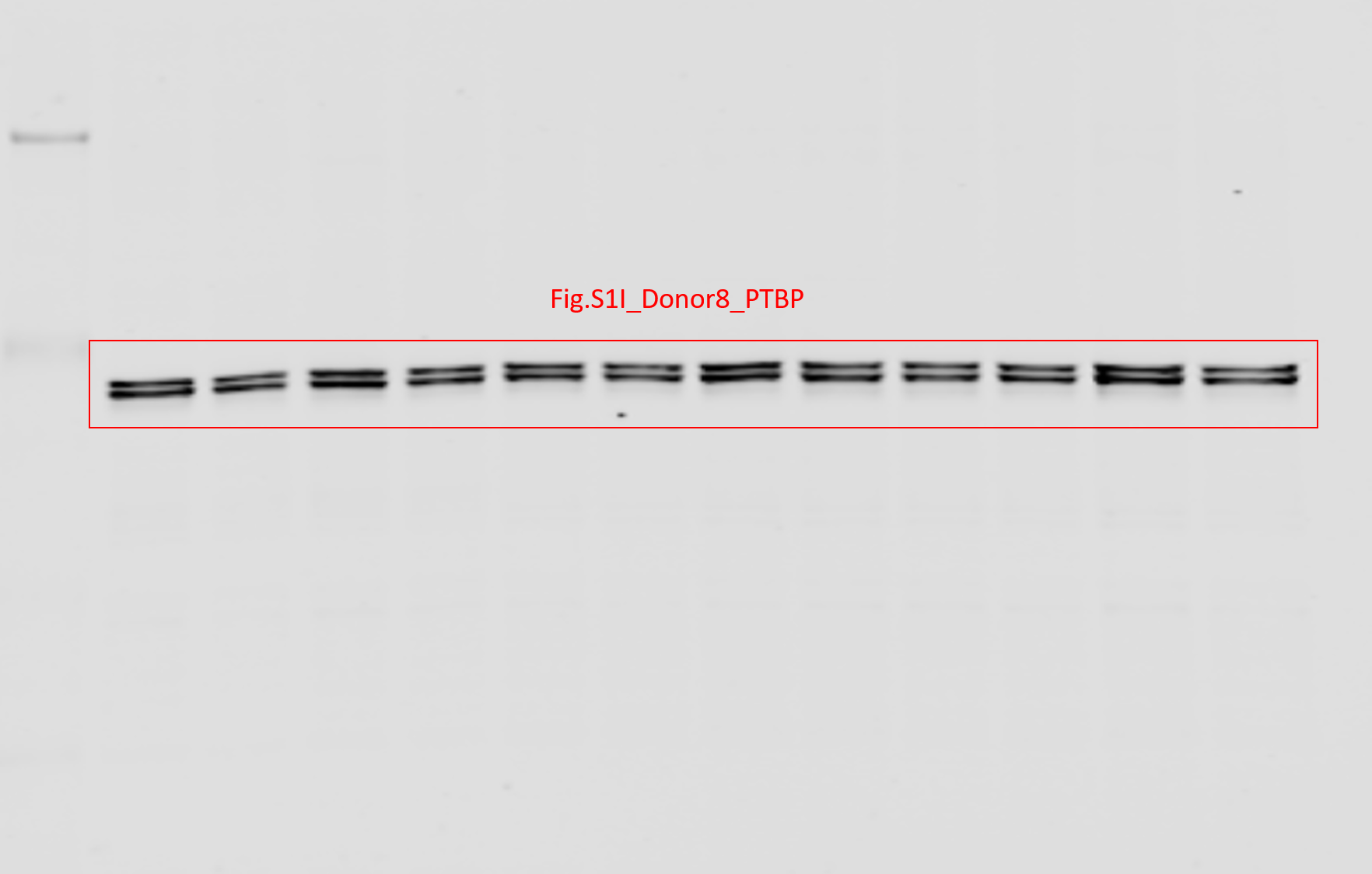

Supplement: Figure 2—source data 1. [file elife-76927-fig2-data1.zip › Fig 2_associated source files/Fig 2E_Source Data 6_Labeled.tif]

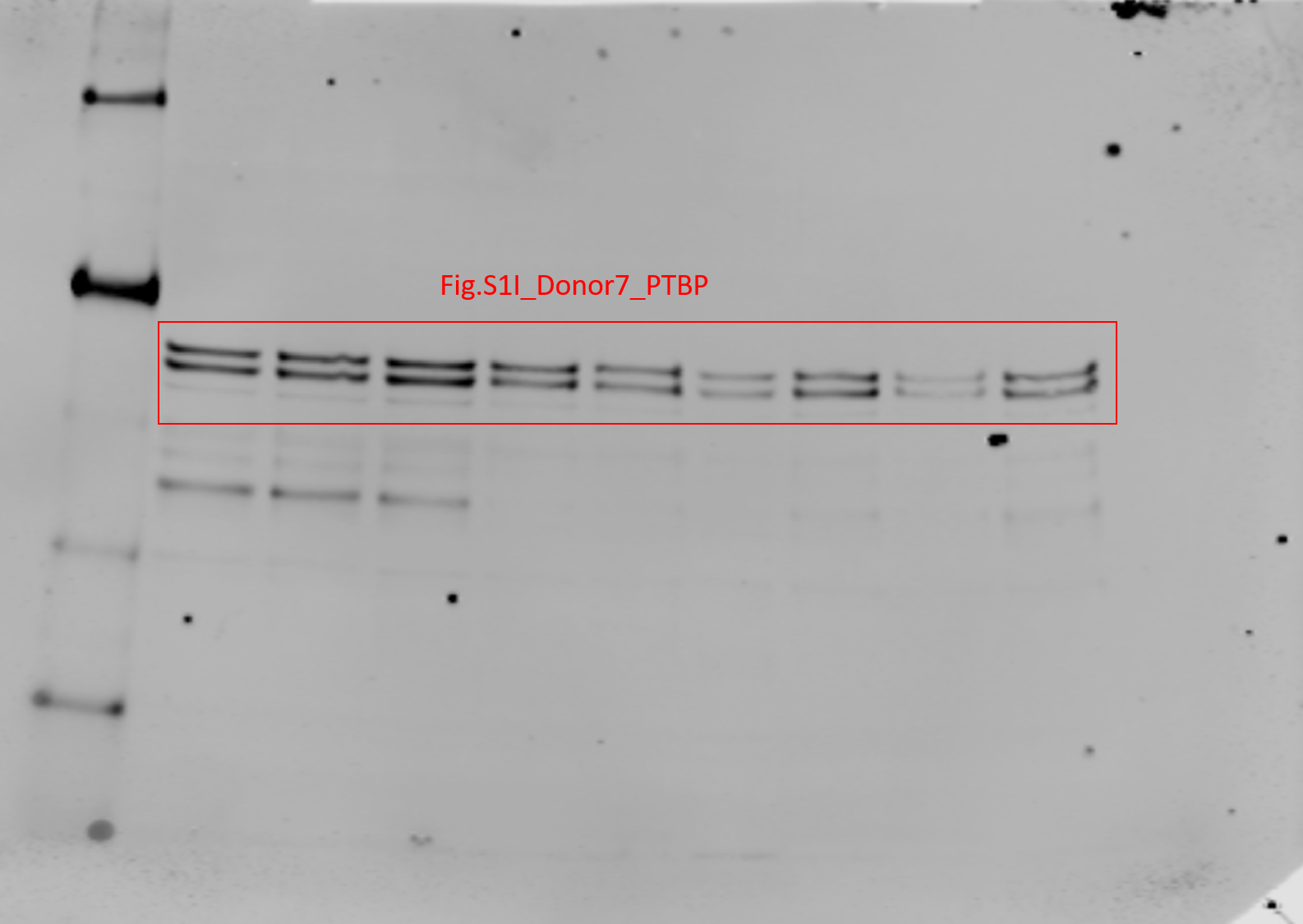

Supplement: Figure 2—source data 1. [file elife-76927-fig2-data1.zip › Fig 2_associated source files/Fig 2E_Source Data 3_Labeled.tif]

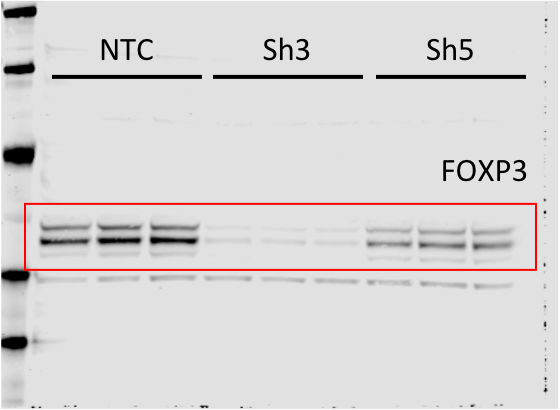

Supplement: Figure 2—source data 1. [file elife-76927-fig2-data1.zip › Fig 2_associated source files/Fig 2C_Source Data 2_Labeled.png]

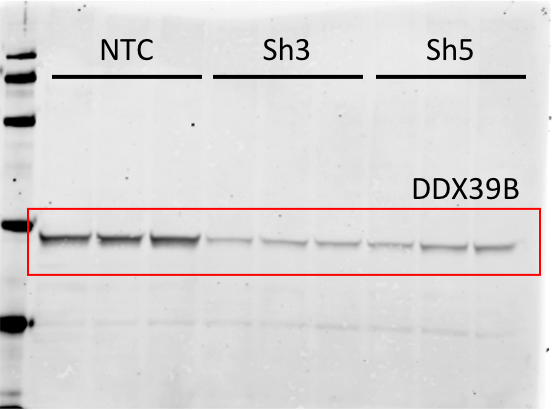

Supplement: Figure 2—source data 1. [file elife-76927-fig2-data1.zip › Fig 2_associated source files/Fig 2C_Source Data 1_Labeled.png]

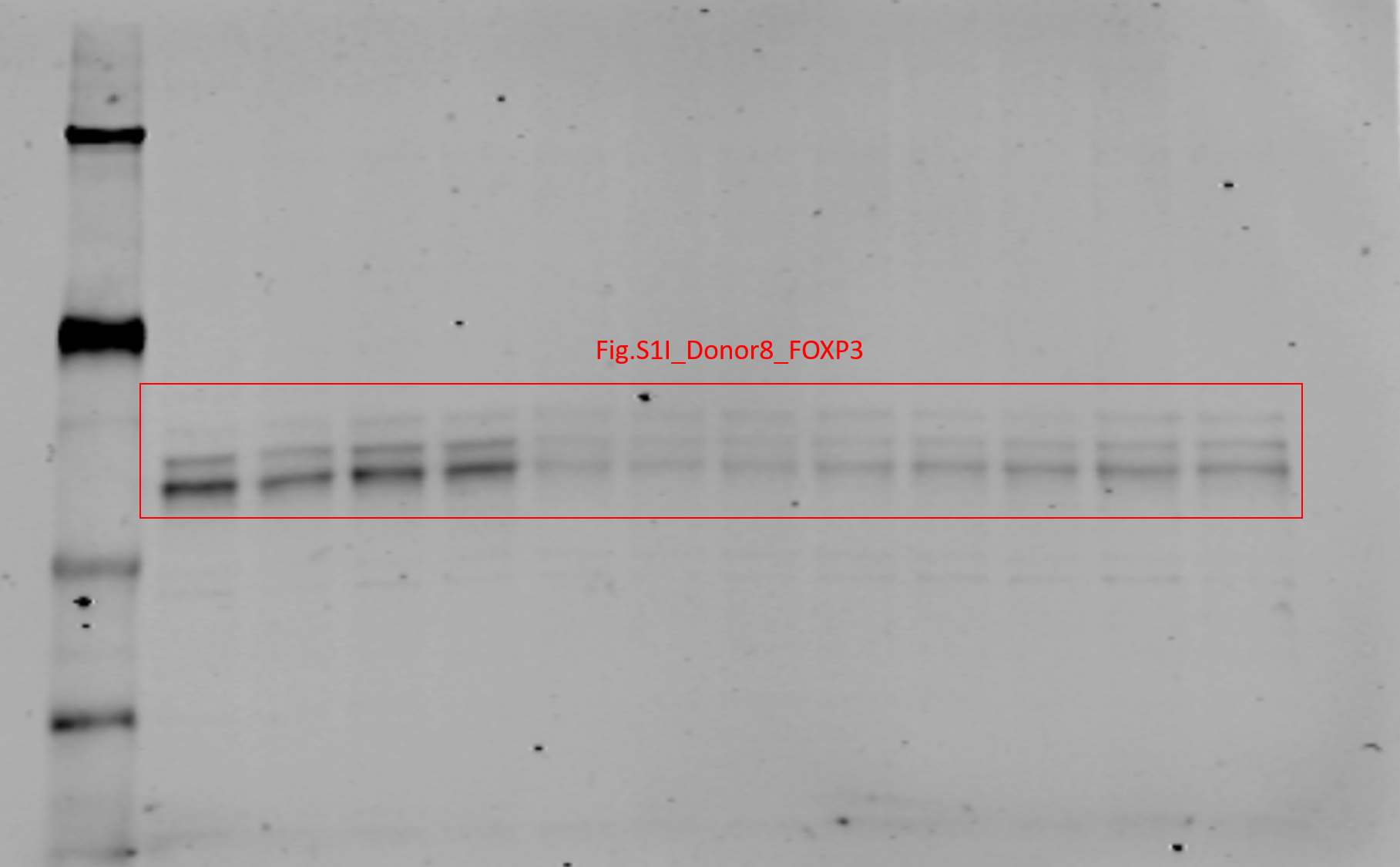

Supplement: Figure 2—source data 1. [file elife-76927-fig2-data1.zip › Fig 2_associated source files/Fig 2E_Source Data 5_Labeled.tif]

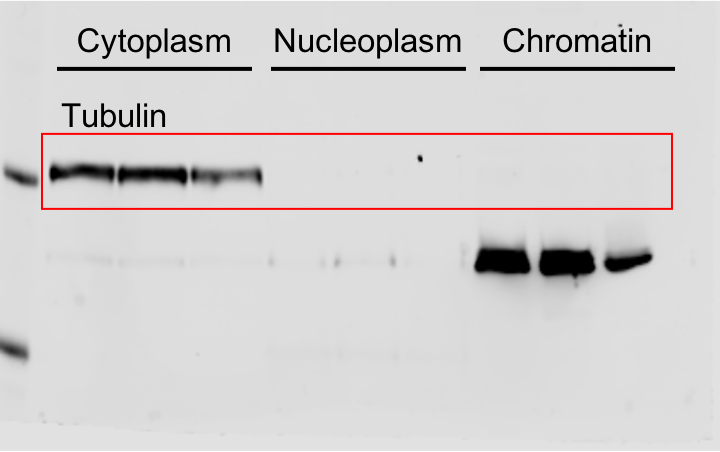

Supplement: Figure 4—source data 1. [file elife-76927-fig4-data1.zip › Fig 4_associated source files/Fig 4A_Source Data 3_Labeled.png]

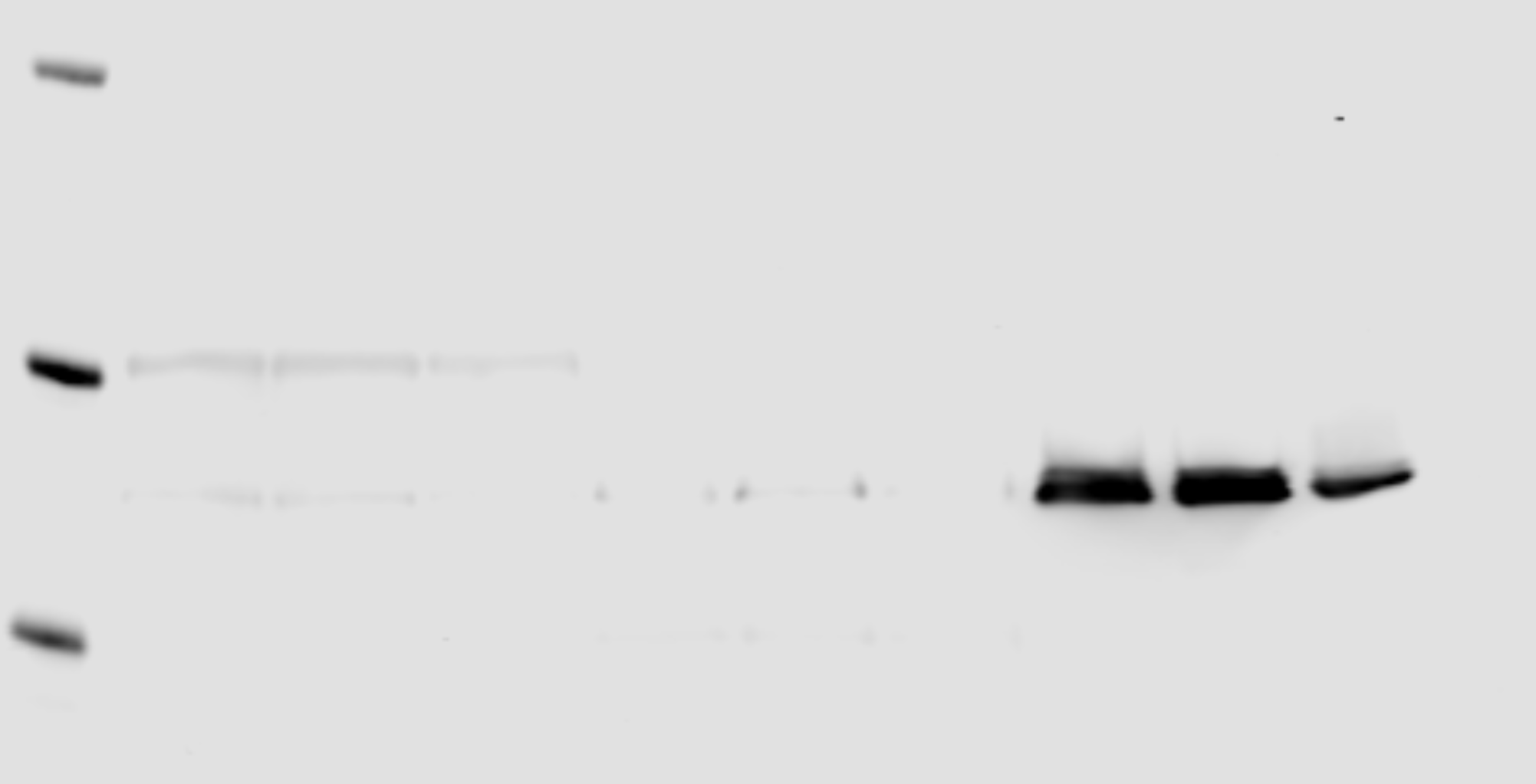

Supplement: Figure 4—source data 1. [file elife-76927-fig4-data1.zip › Fig 4_associated source files/Fig 4A_Source Data 1.tif]

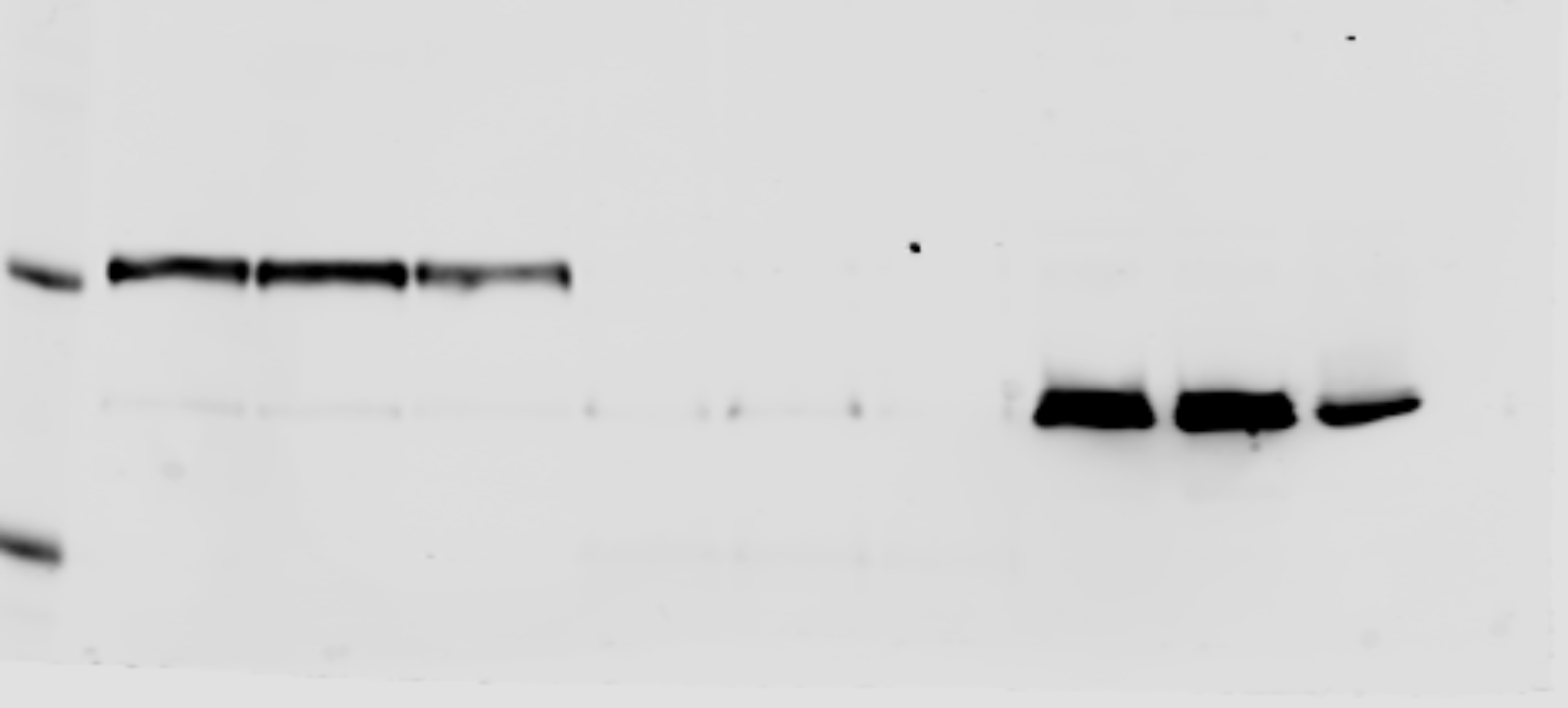

Supplement: Figure 4—source data 1. [file elife-76927-fig4-data1.zip › Fig 4_associated source files/Fig 4A_Source Data 3.tif]

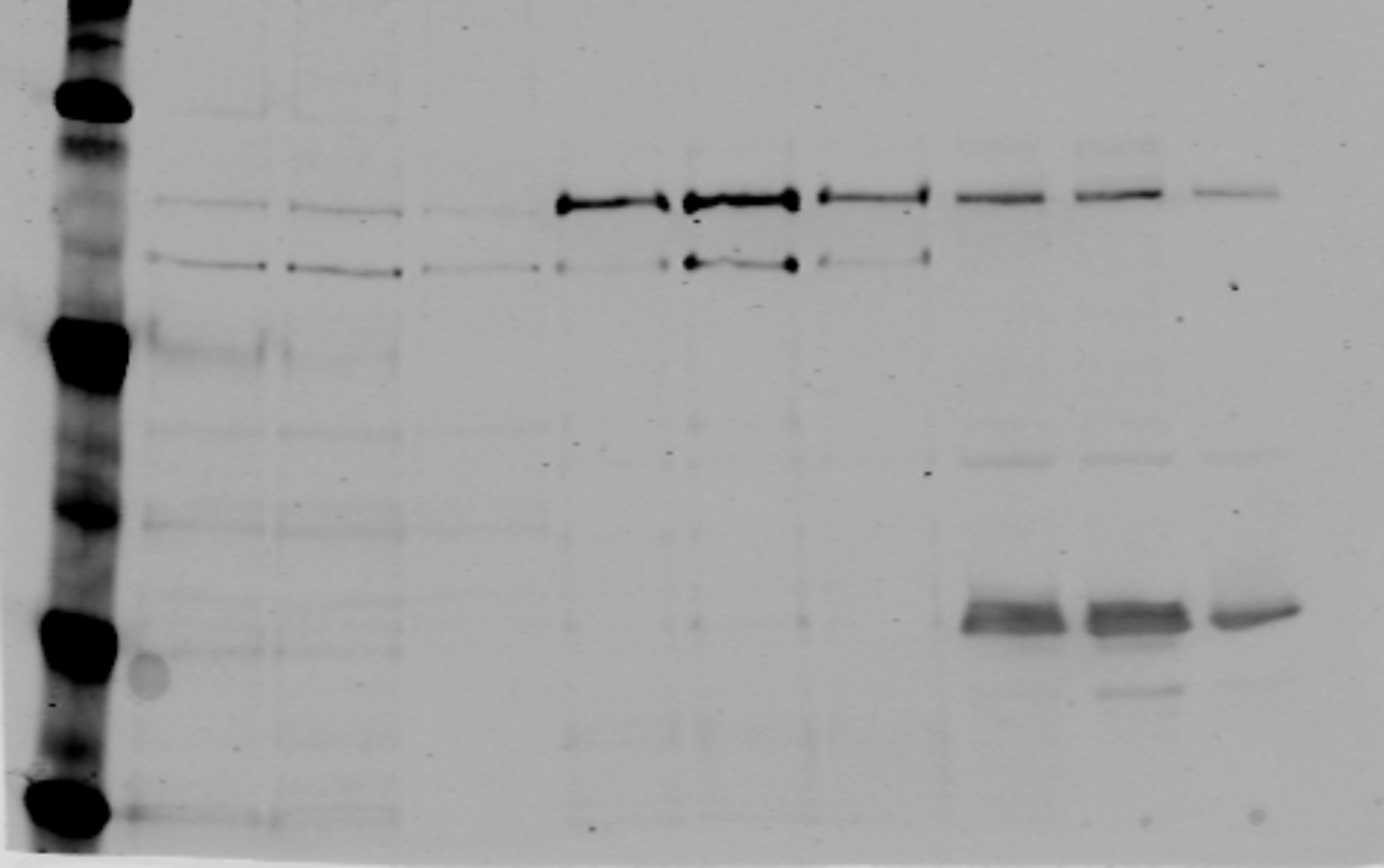

Supplement: Figure 4—source data 1. [file elife-76927-fig4-data1.zip › Fig 4_associated source files/Fig 4A_Source Data 2.tif]

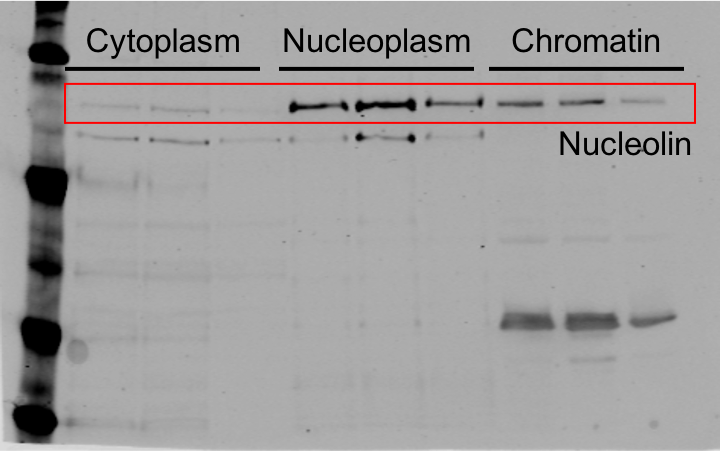

Supplement: Figure 4—source data 1. [file elife-76927-fig4-data1.zip › Fig 4_associated source files/Fig 4A_Source Data 2_Labeled.png]

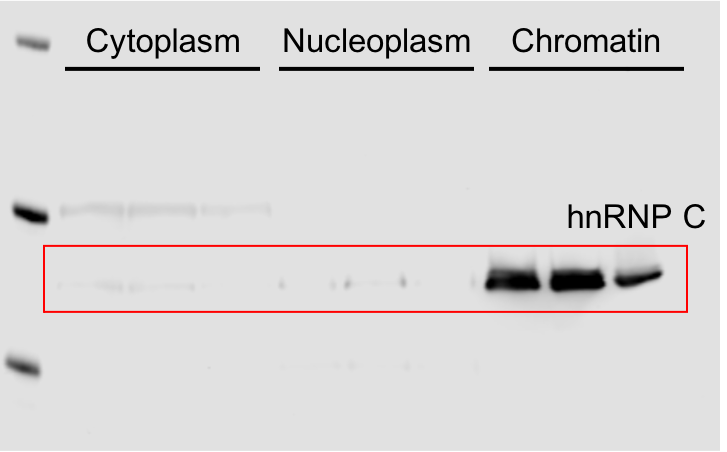

Supplement: Figure 4—source data 1. [file elife-76927-fig4-data1.zip › Fig 4_associated source files/Fig 4A_Source Data 1_Labeled.png]

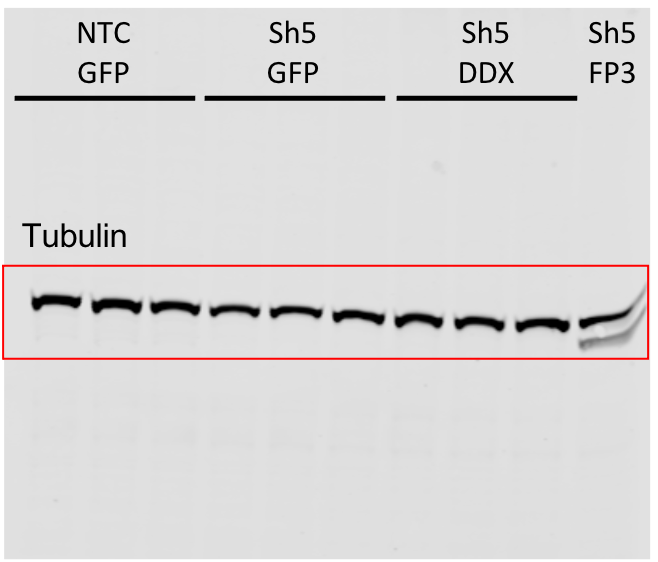

Supplement: Figure 5—source data 1. [file elife-76927-fig5-data1.zip › Fig 5_associated source files/Fig 5D_Source Data 3_Labeled.png]

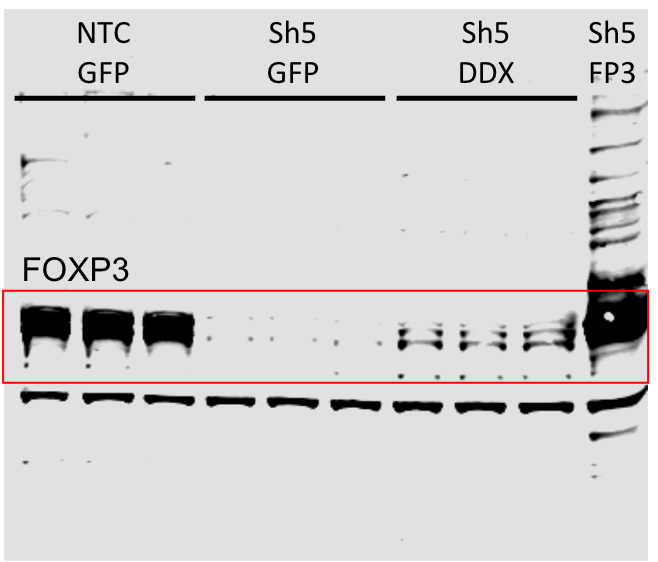

Supplement: Figure 5—source data 1. [file elife-76927-fig5-data1.zip › Fig 5_associated source files/Fig 5D_Source Data 2_Labeled.png]

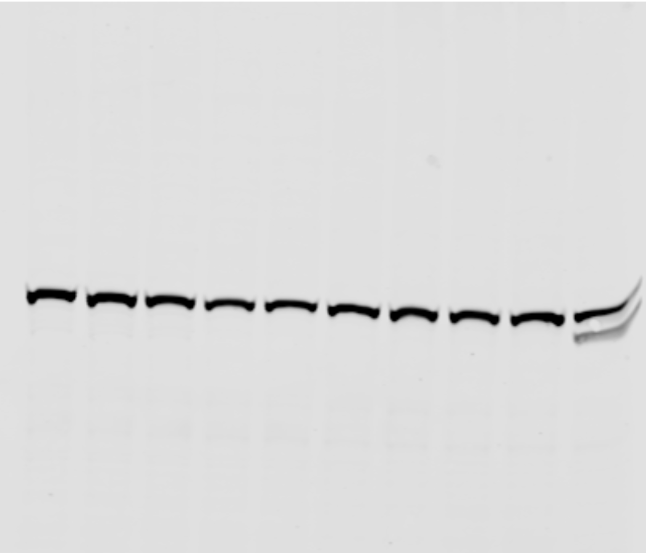

Supplement: Figure 5—source data 1. [file elife-76927-fig5-data1.zip › Fig 5_associated source files/Fig 5D_Source Data 3.png]

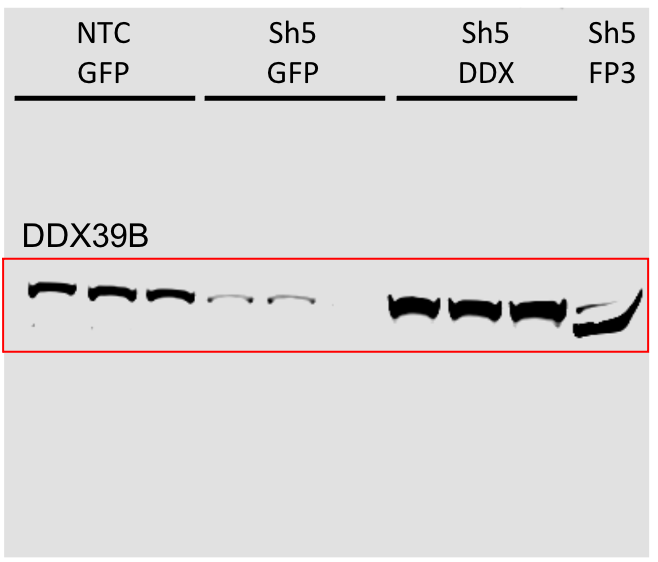

Supplement: Figure 5—source data 1. [file elife-76927-fig5-data1.zip › Fig 5_associated source files/Fig 5D_Source Data 1_Labeled.png]

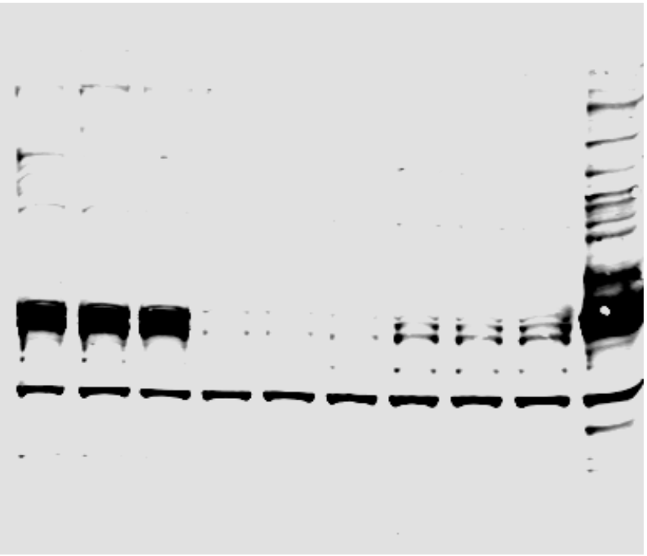

Supplement: Figure 5—source data 1. [file elife-76927-fig5-data1.zip › Fig 5_associated source files/Fig 5D_Source Data 2.png]

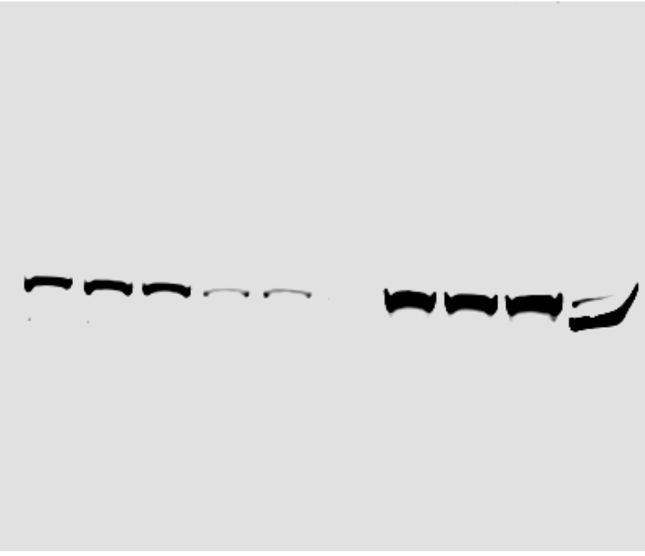

Supplement: Figure 5—source data 1. [file elife-76927-fig5-data1.zip › Fig 5_associated source files/Fig 5D_Source Data 1.png]

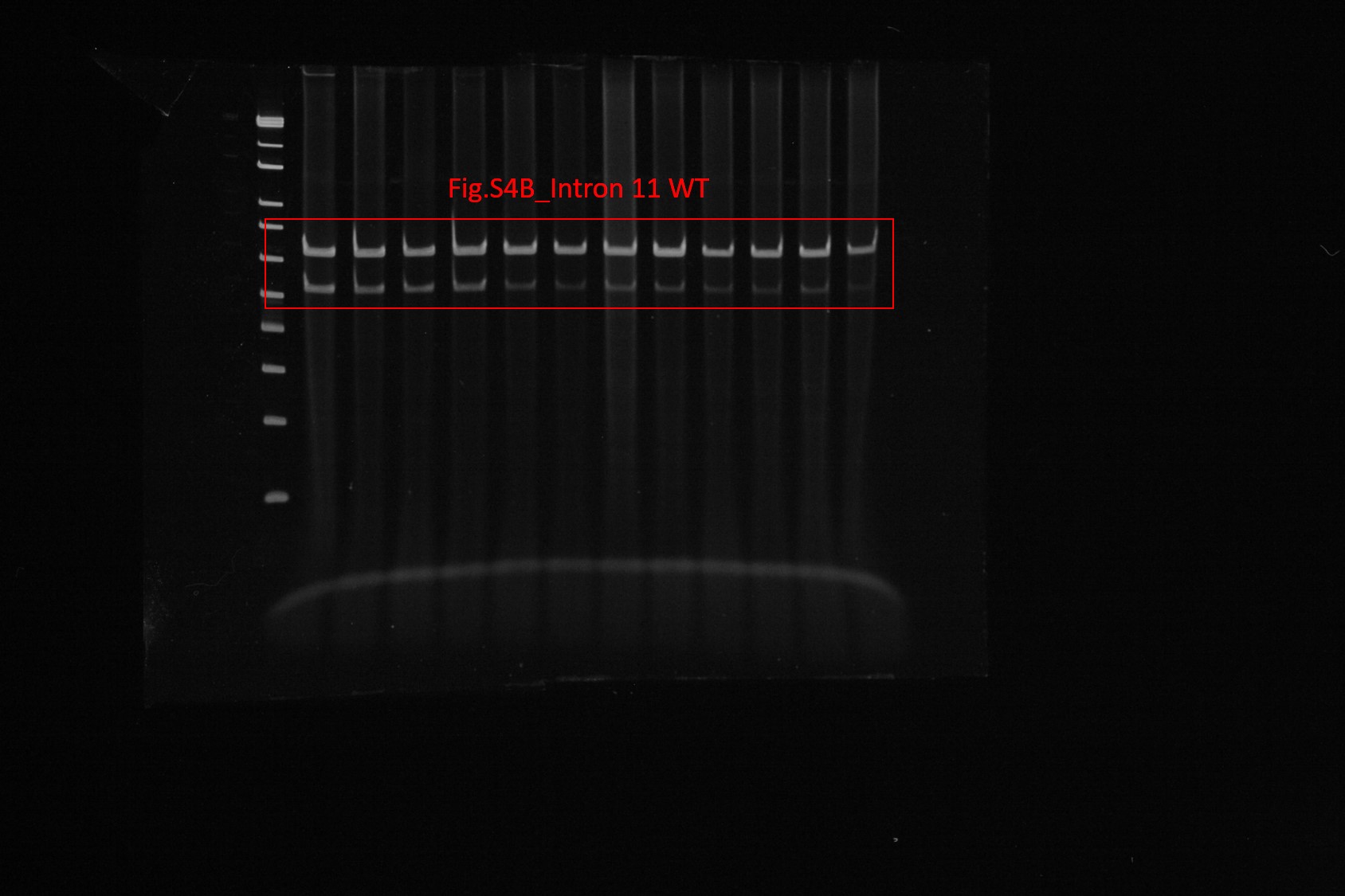

Supplement: Figure 7—figure supplement 2—source data 1. [file elife-76927-fig7-figsupp2-data1.zip › Fig 7_figure supplement 2_associated source files/Fig 7_figure supplement 2_source data 1_Labeled.jpg]

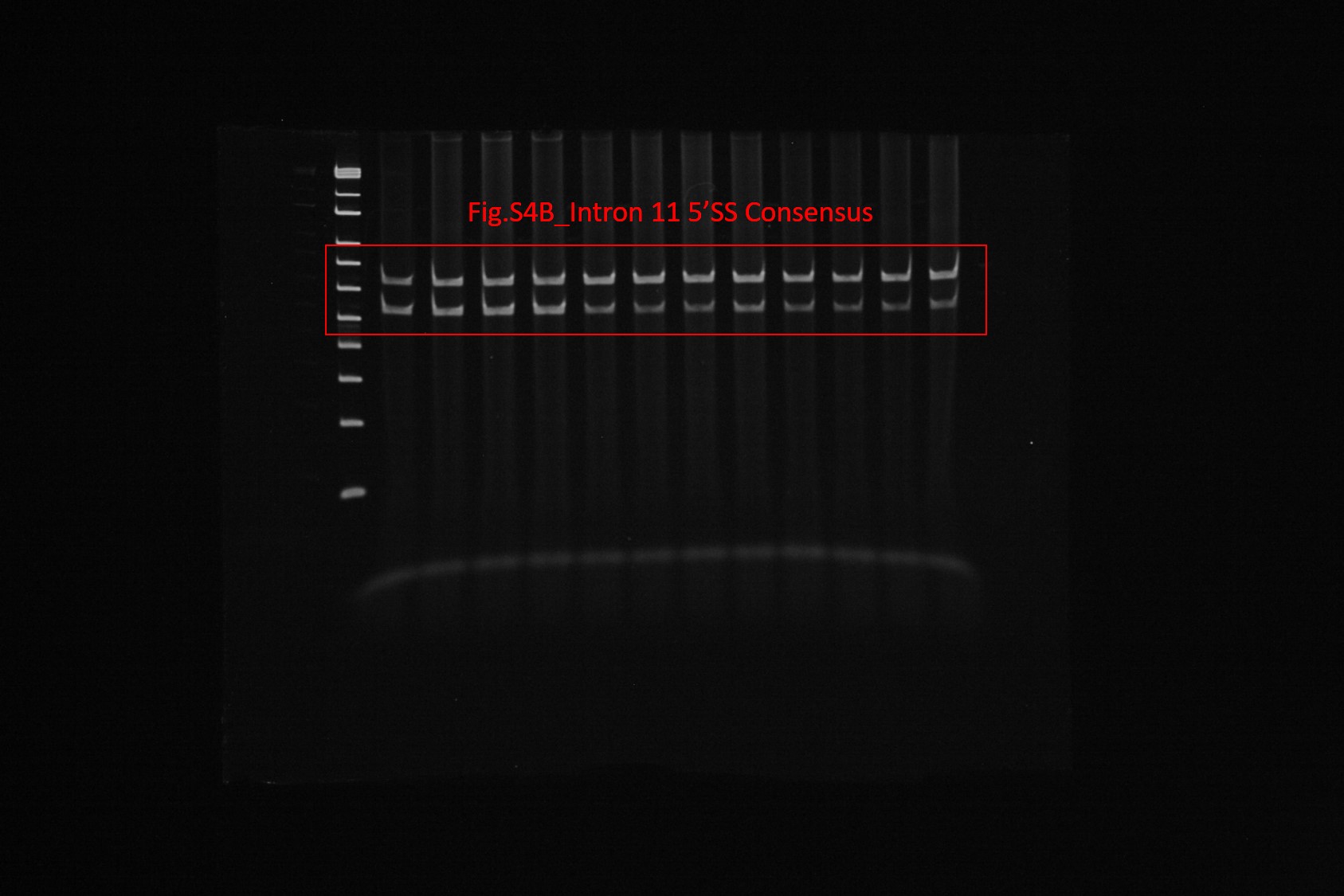

Supplement: Figure 7—figure supplement 2—source data 1. [file elife-76927-fig7-figsupp2-data1.zip › Fig 7_figure supplement 2_associated source files/Fig 7_figure supplement 2_source data 2_Labeled.jpg]

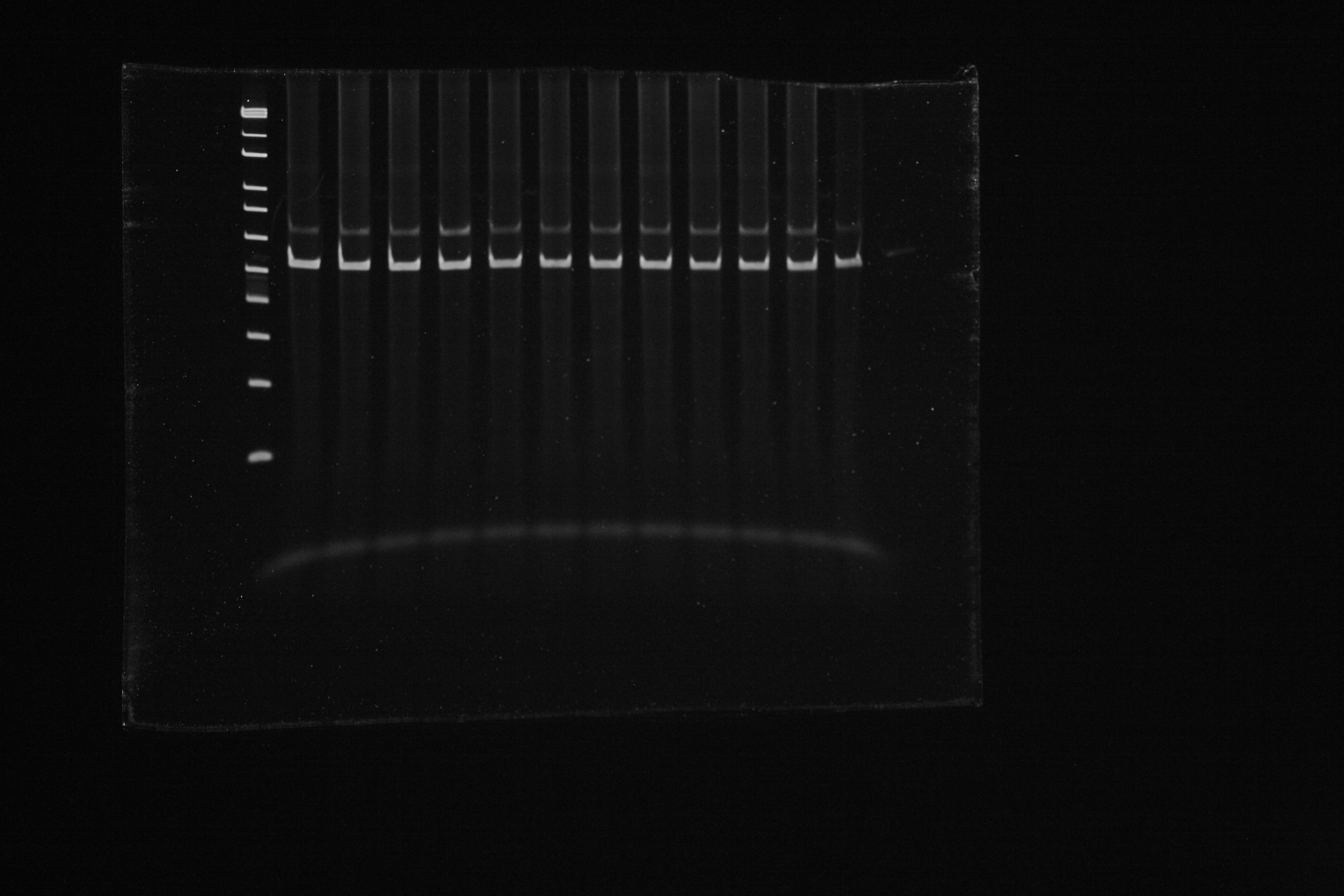

Supplement: Figure 7—figure supplement 2—source data 1. [file elife-76927-fig7-figsupp2-data1.zip › Fig 7_figure supplement 2_associated source files/Fig 7_figure supplement 2_source data 3.JPG]

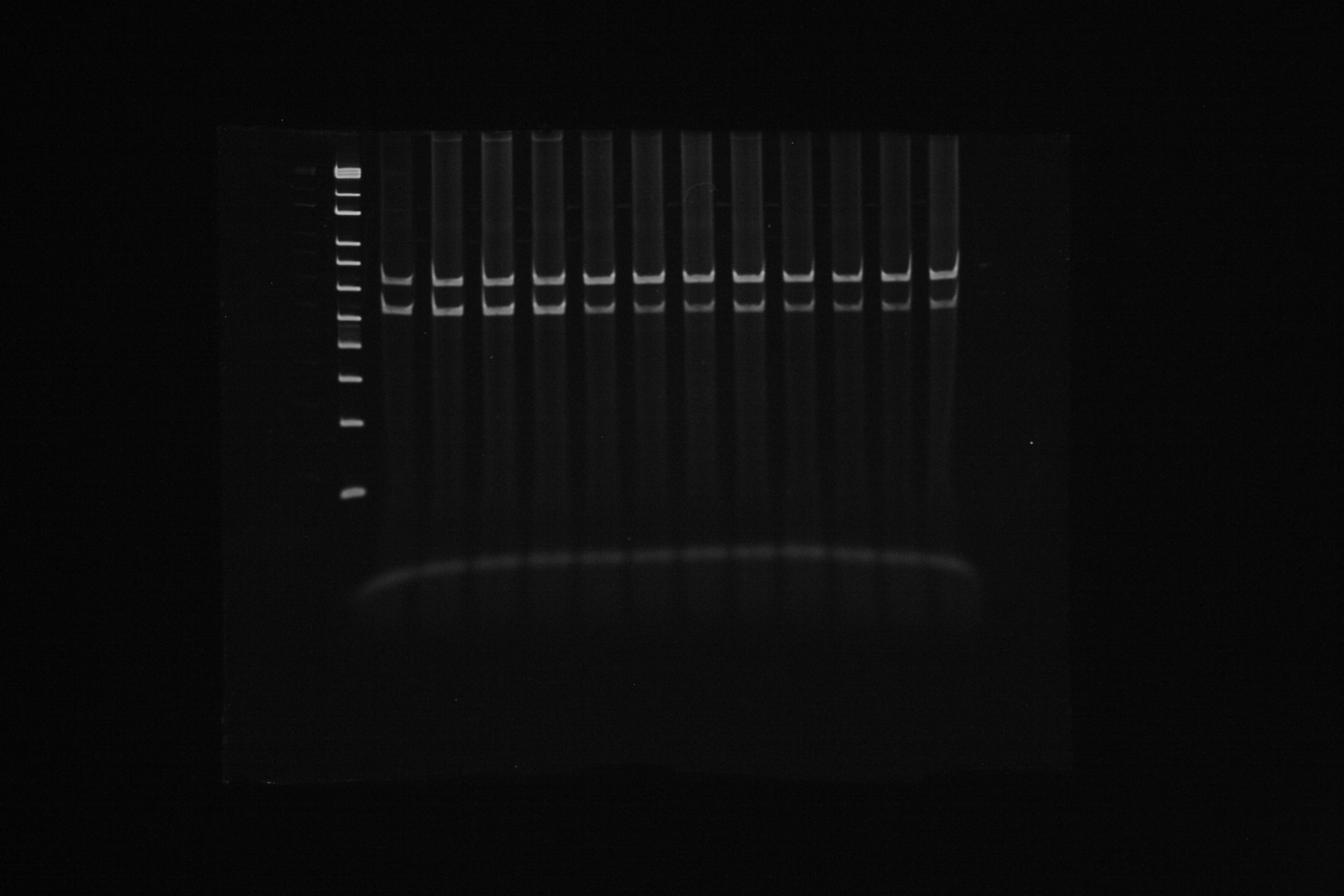

Supplement: Figure 7—figure supplement 2—source data 1. [file elife-76927-fig7-figsupp2-data1.zip › Fig 7_figure supplement 2_associated source files/Fig 7_figure supplement 2_source data 2.JPG]

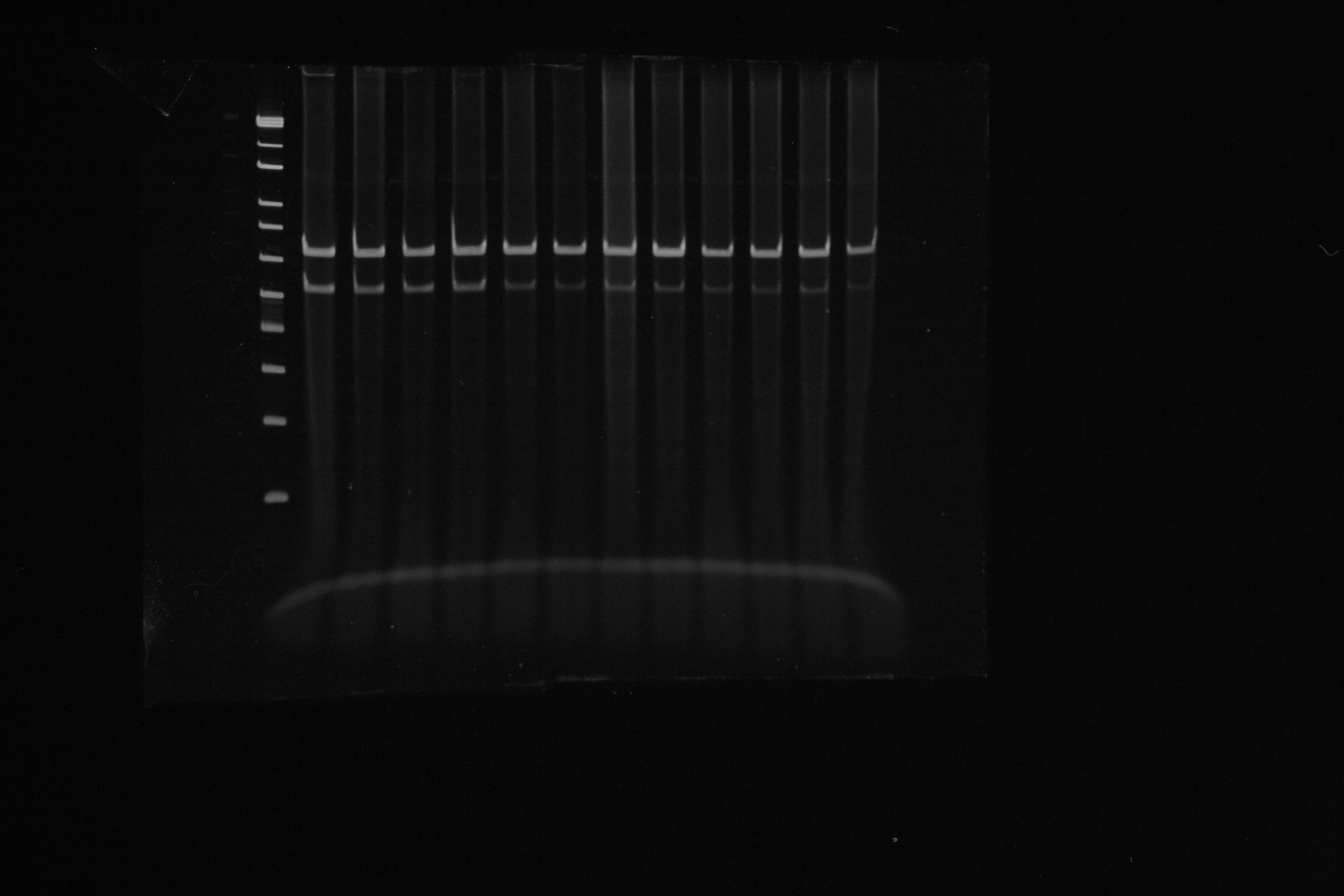

Supplement: Figure 7—figure supplement 2—source data 1. [file elife-76927-fig7-figsupp2-data1.zip › Fig 7_figure supplement 2_associated source files/Fig 7_figure supplement 2_source data 1.JPG]

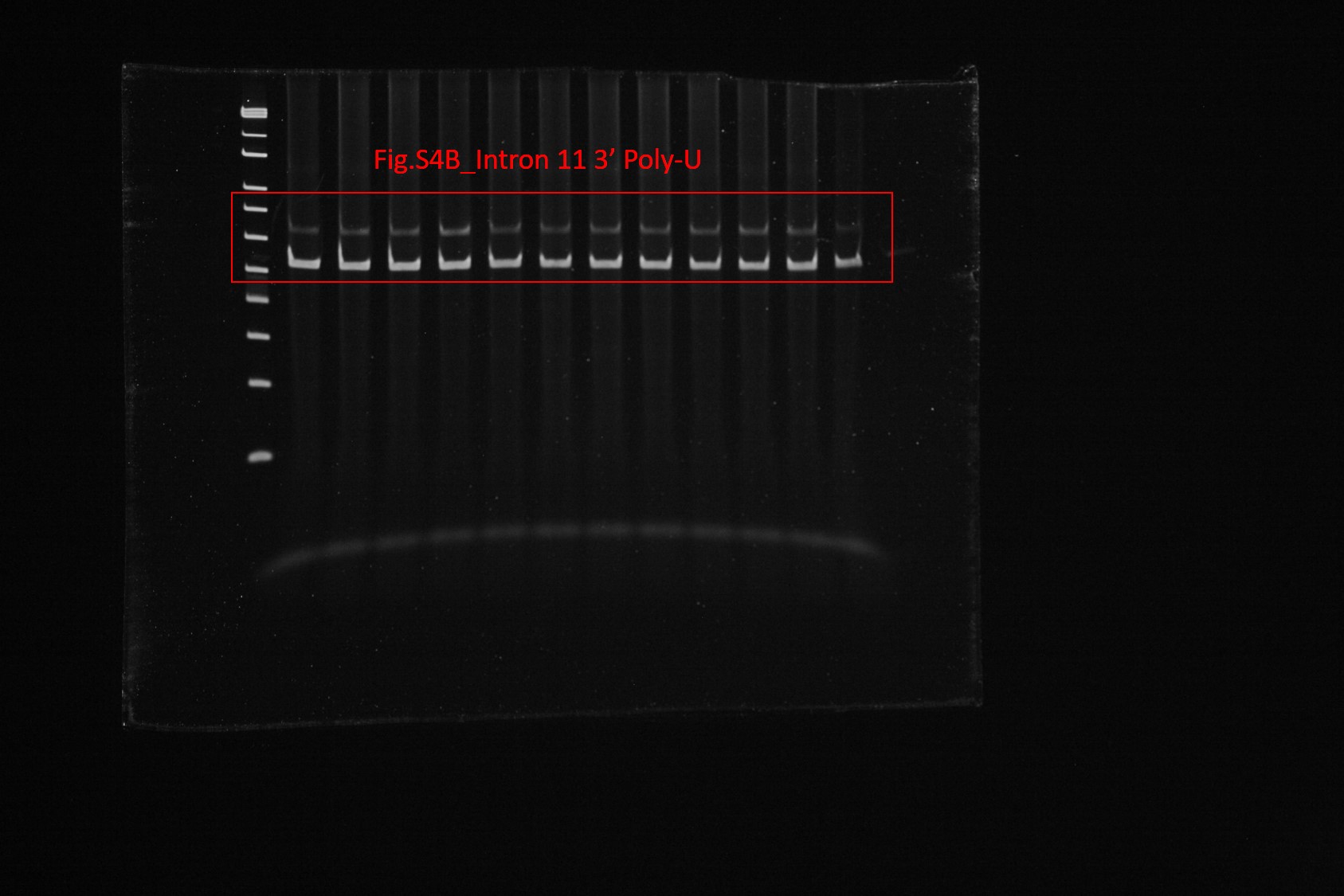

Supplement: Figure 7—figure supplement 2—source data 1. [file elife-76927-fig7-figsupp2-data1.zip › Fig 7_figure supplement 2_associated source files/Fig 7_figure supplement 2_source data 3_Labeled.jpg]

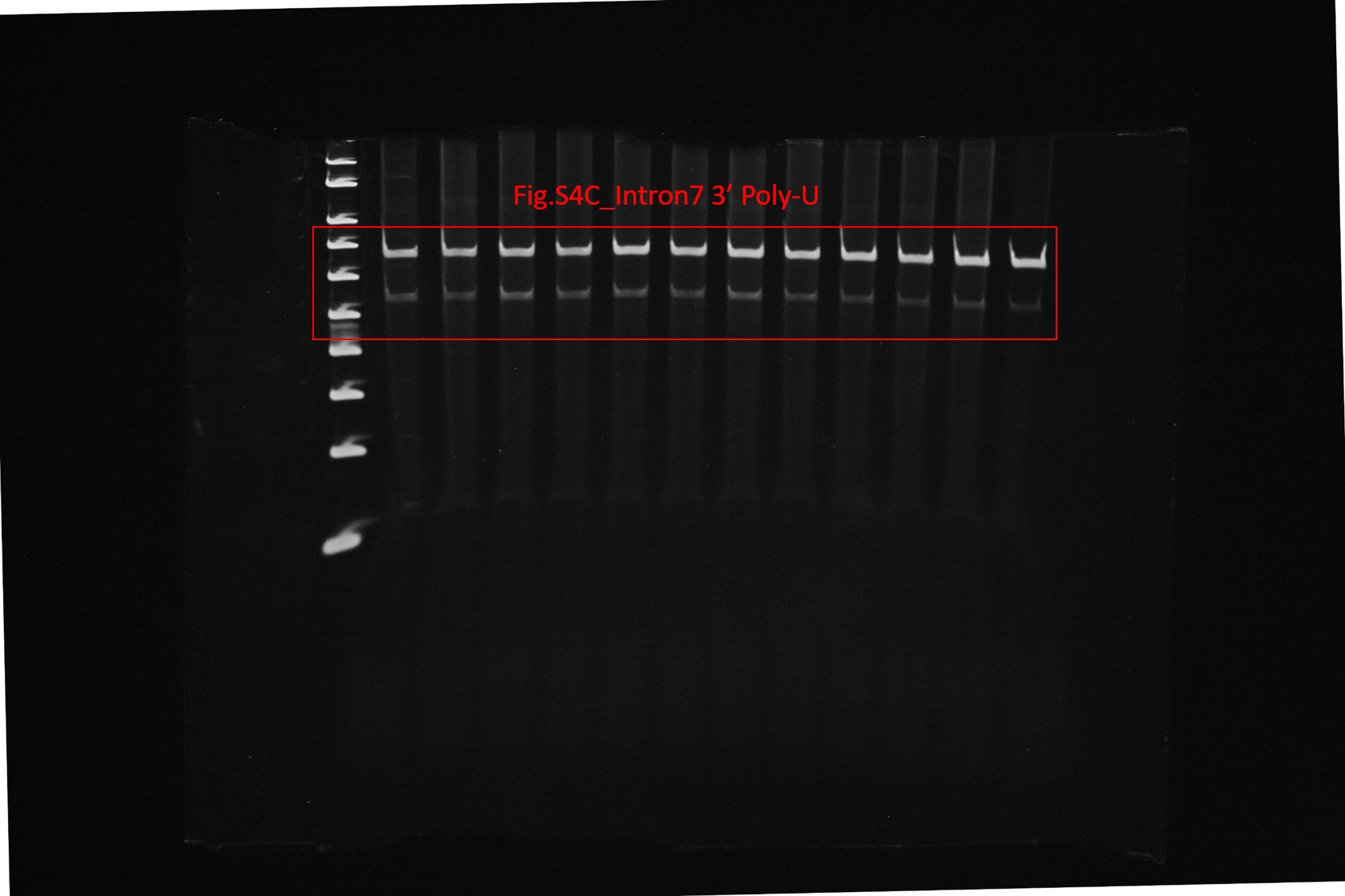

Supplement: Figure 7—figure supplement 3—source data 1. [file elife-76927-fig7-figsupp3-data1.zip › Fig 7_figure supplement 3_associated source files/Fig 7_figure supplement 3_source data 3_Labeled.jpg]

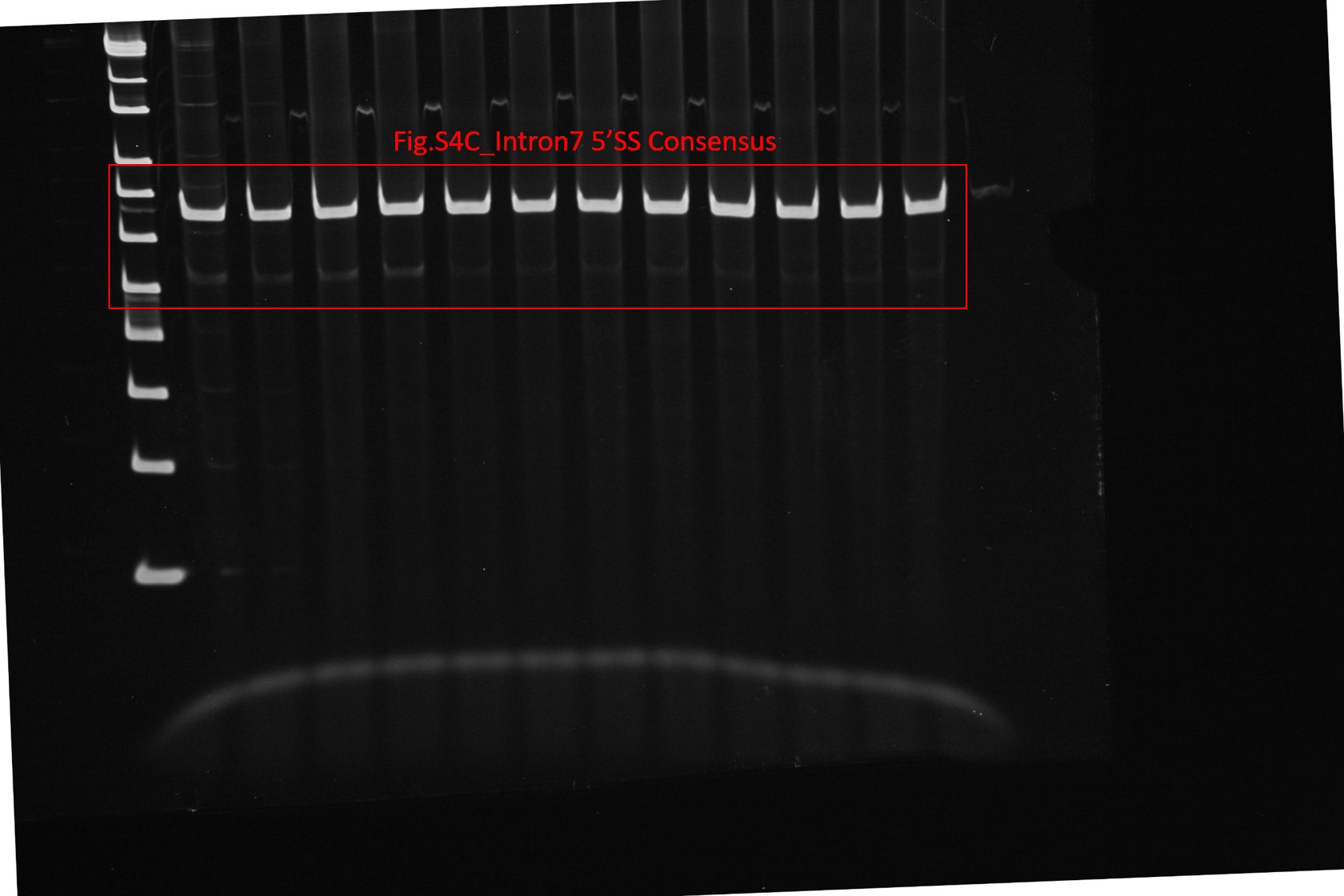

Supplement: Figure 7—figure supplement 3—source data 1. [file elife-76927-fig7-figsupp3-data1.zip › Fig 7_figure supplement 3_associated source files/Fig 7_figure supplement 3_source data 2_Labeled.jpg]

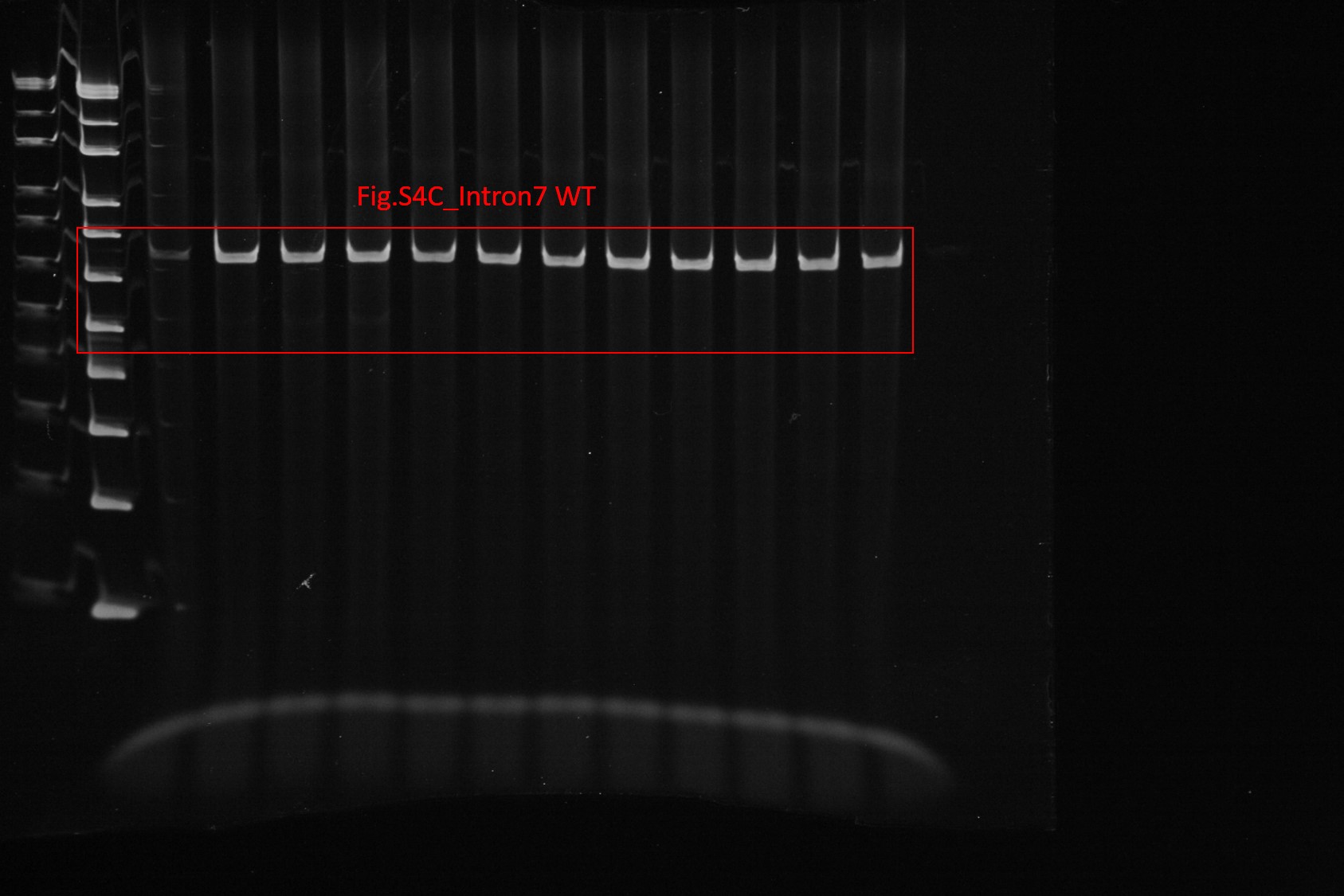

Supplement: Figure 7—figure supplement 3—source data 1. [file elife-76927-fig7-figsupp3-data1.zip › Fig 7_figure supplement 3_associated source files/Fig 7_figure supplement 3_source data 1_Labeled.jpg]

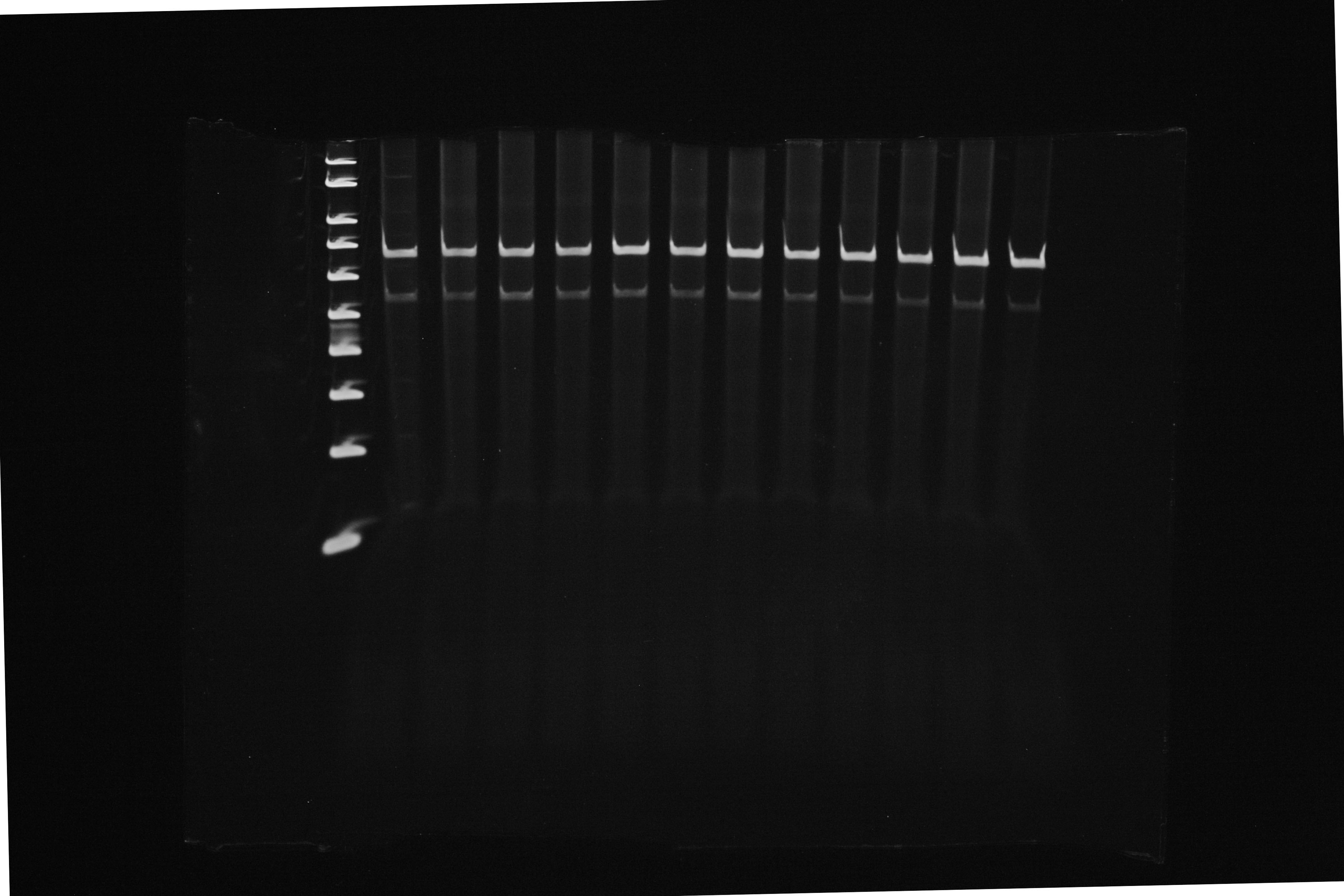

Supplement: Figure 7—figure supplement 3—source data 1. [file elife-76927-fig7-figsupp3-data1.zip › Fig 7_figure supplement 3_associated source files/Fig 7_figure supplement 3_source data 3.jpg]

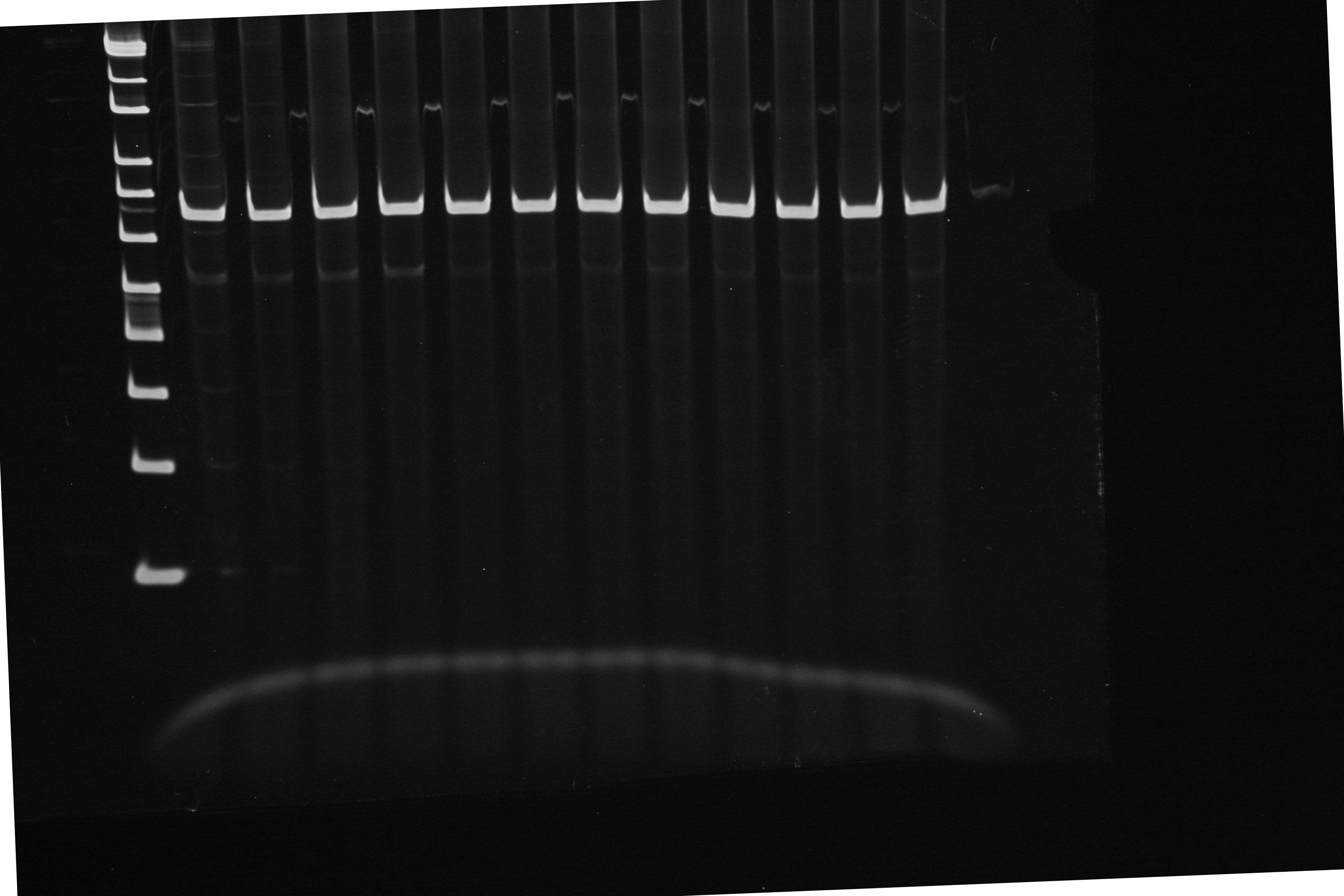

Supplement: Figure 7—figure supplement 3—source data 1. [file elife-76927-fig7-figsupp3-data1.zip › Fig 7_figure supplement 3_associated source files/Fig 7_figure supplement 3_source data 2.jpg]

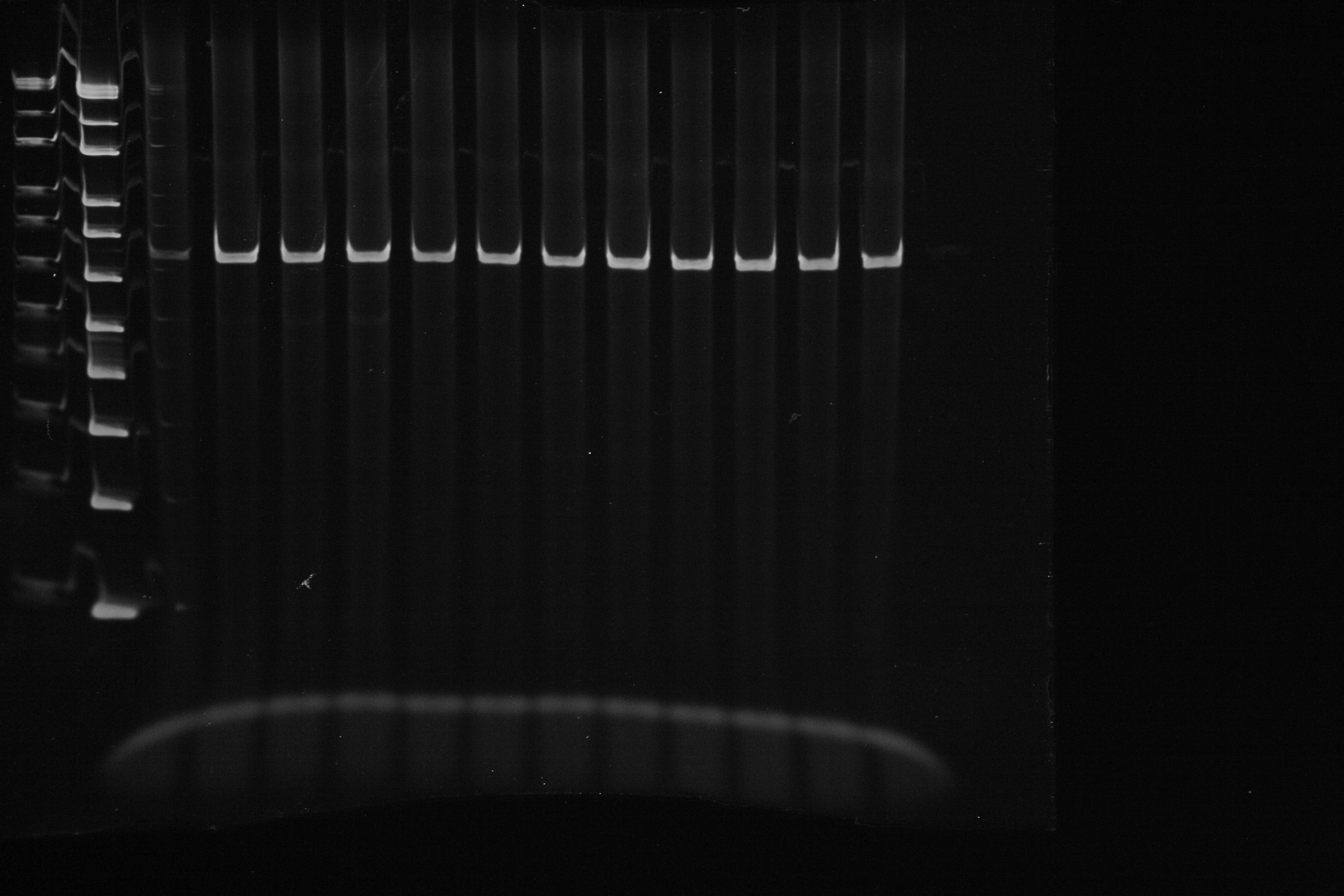

Supplement: Figure 7—figure supplement 3—source data 1. [file elife-76927-fig7-figsupp3-data1.zip › Fig 7_figure supplement 3_associated source files/Fig 7_figure supplement 3_source data 1.JPG]

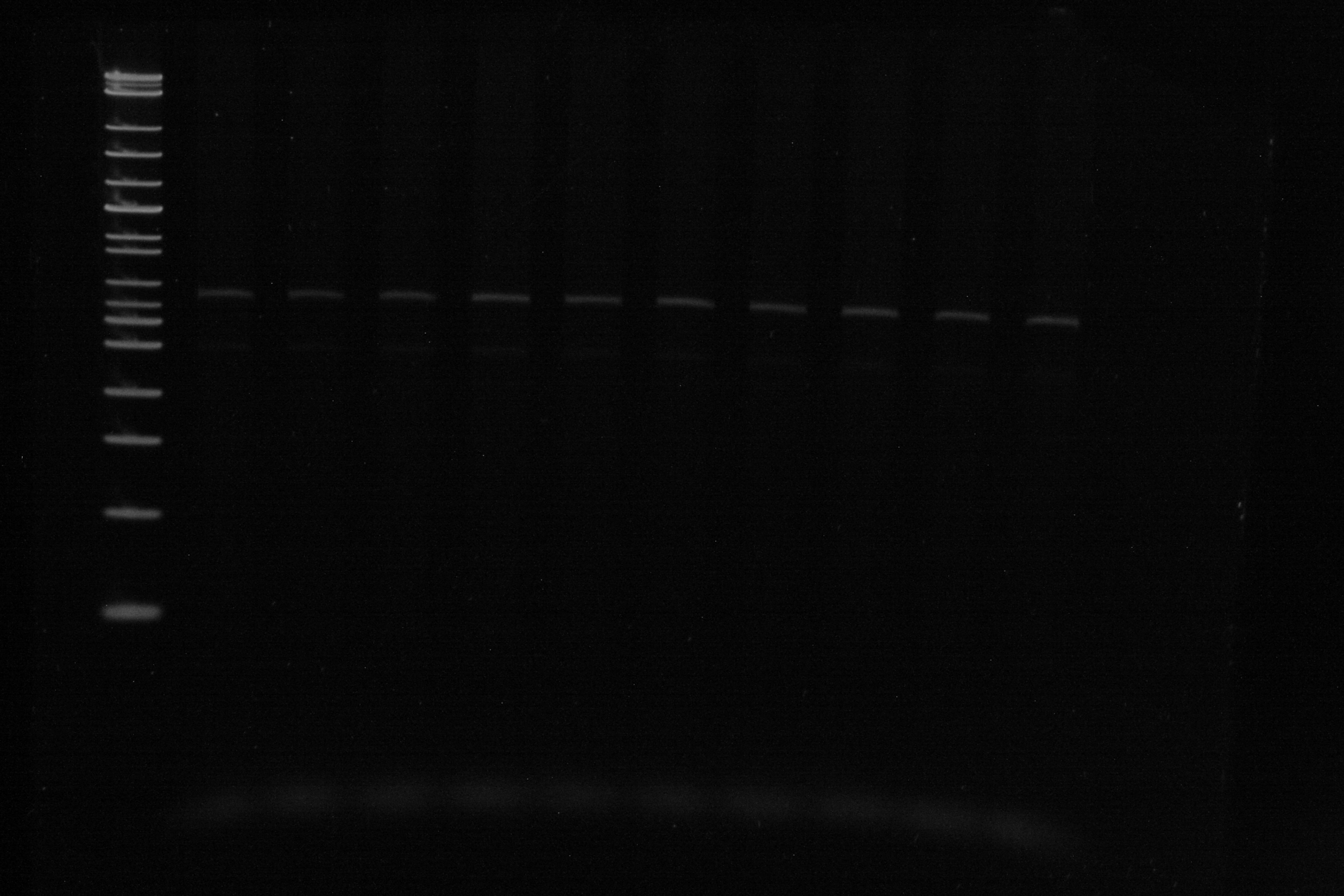

Supplement: Figure 7—figure supplement 8—source data 1. [file elife-76927-fig7-figsupp8-data1.zip › Fig 7_figure supplement 8_associated source files/Fig 7_figure supplement 8_source data 1.tif]

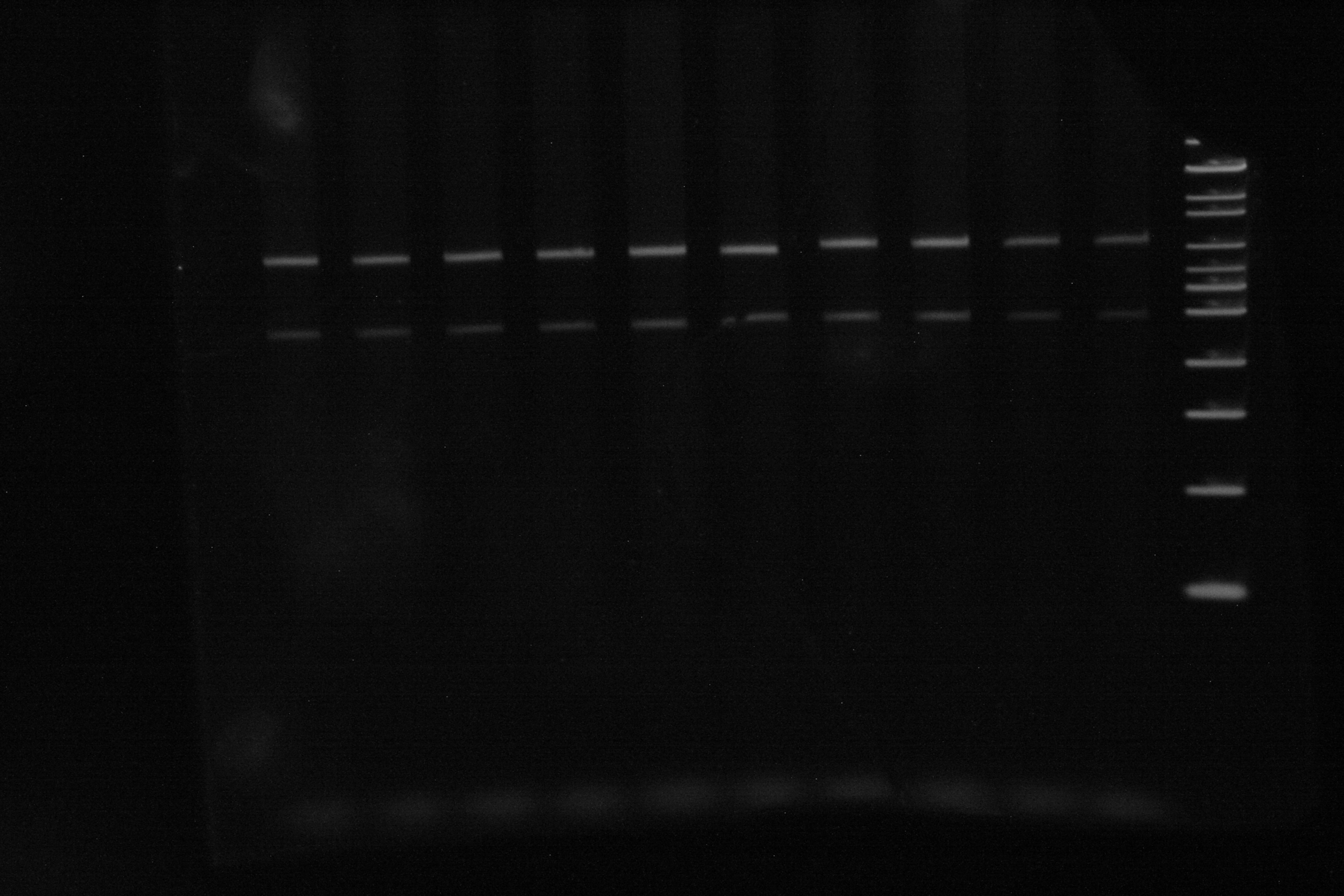

Supplement: Figure 7—figure supplement 8—source data 1. [file elife-76927-fig7-figsupp8-data1.zip › Fig 7_figure supplement 8_associated source files/Fig 7_figure supplement 8_source data 2.tif]

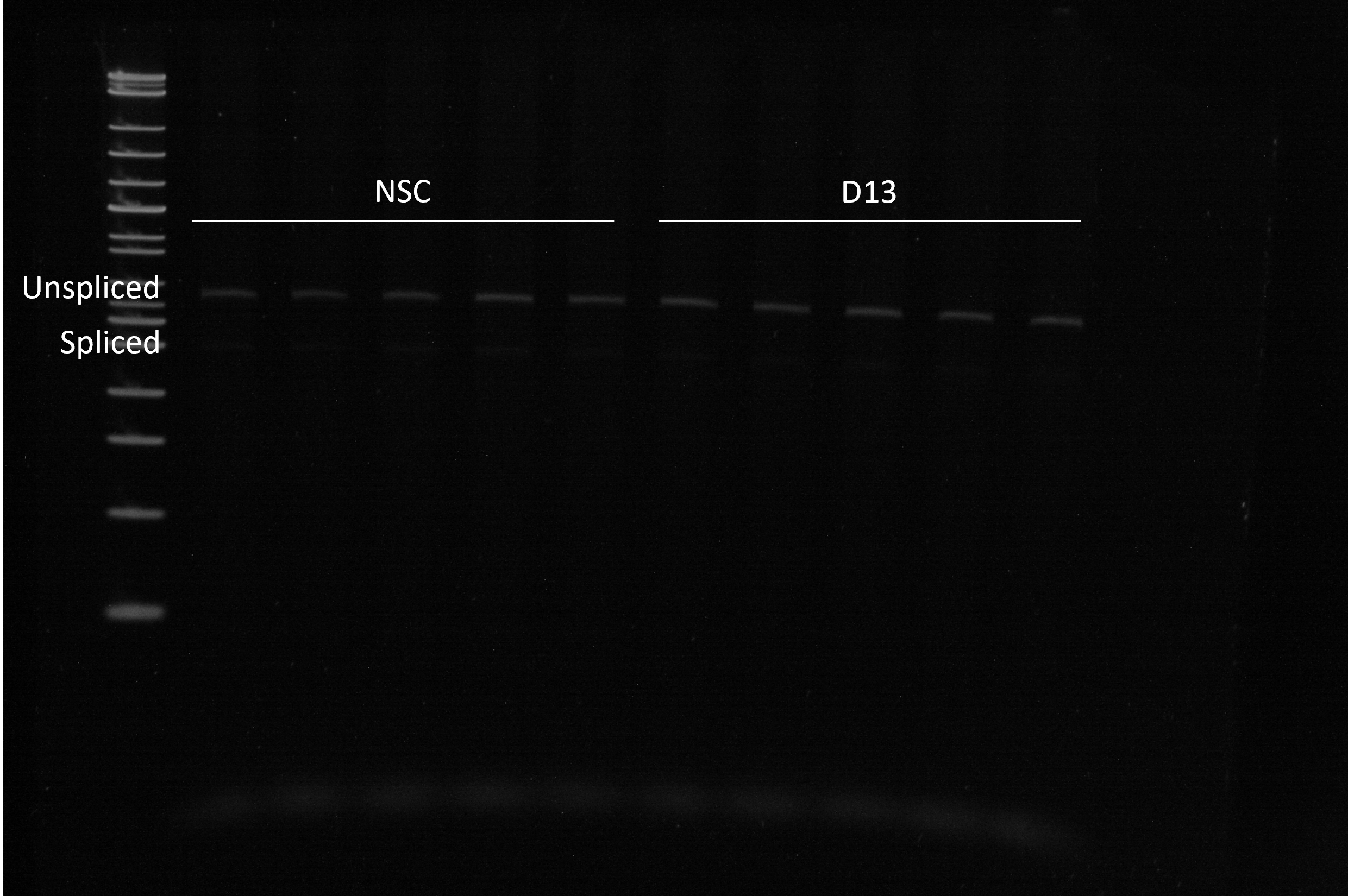

Supplement: Figure 7—figure supplement 8—source data 1. [file elife-76927-fig7-figsupp8-data1.zip › Fig 7_figure supplement 8_associated source files/Fig 7_figure supplement 8_source data 1_labeled.tif]

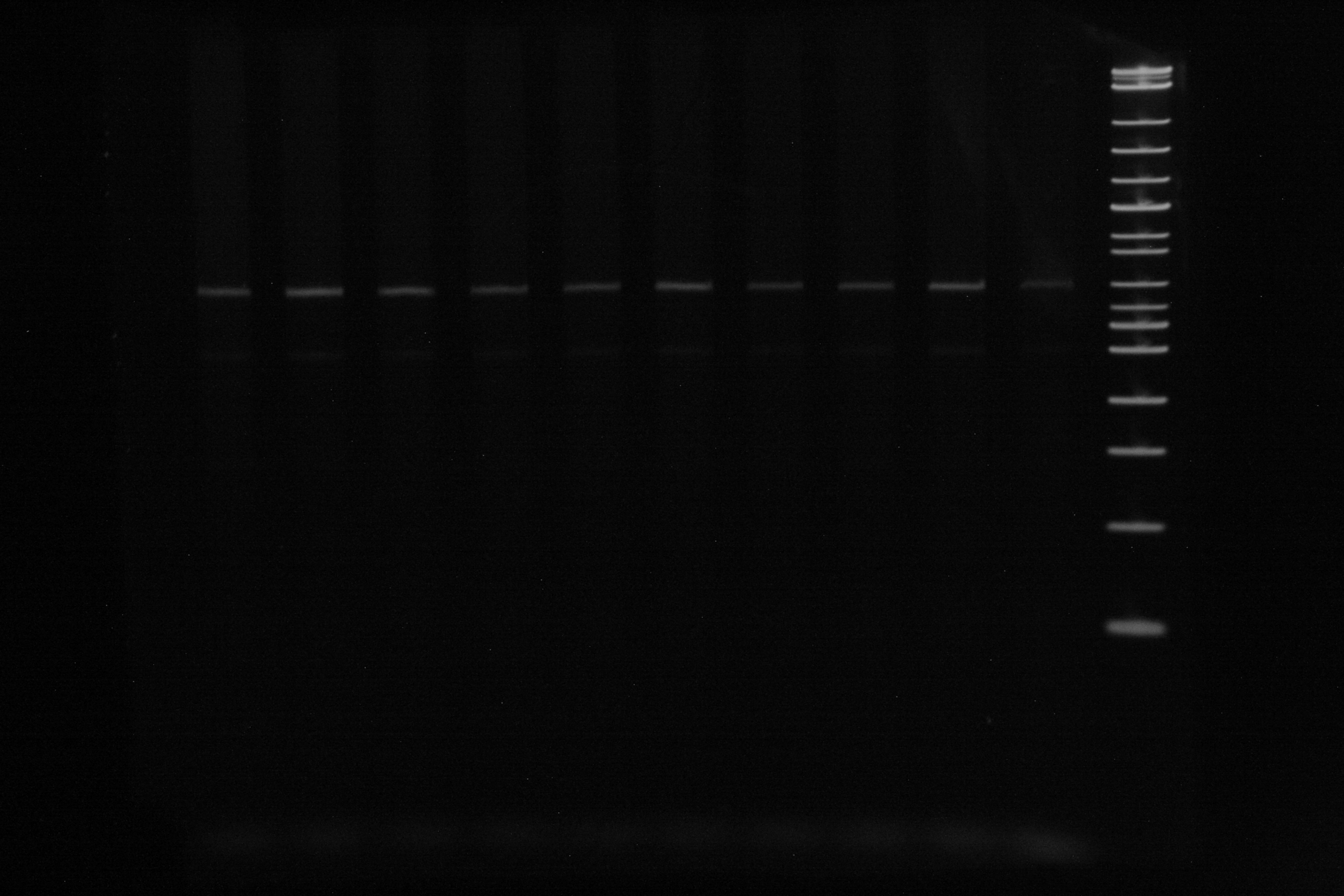

Supplement: Figure 7—figure supplement 8—source data 1. [file elife-76927-fig7-figsupp8-data1.zip › Fig 7_figure supplement 8_associated source files/Fig 7_figure supplement 8_source data 3.tif]

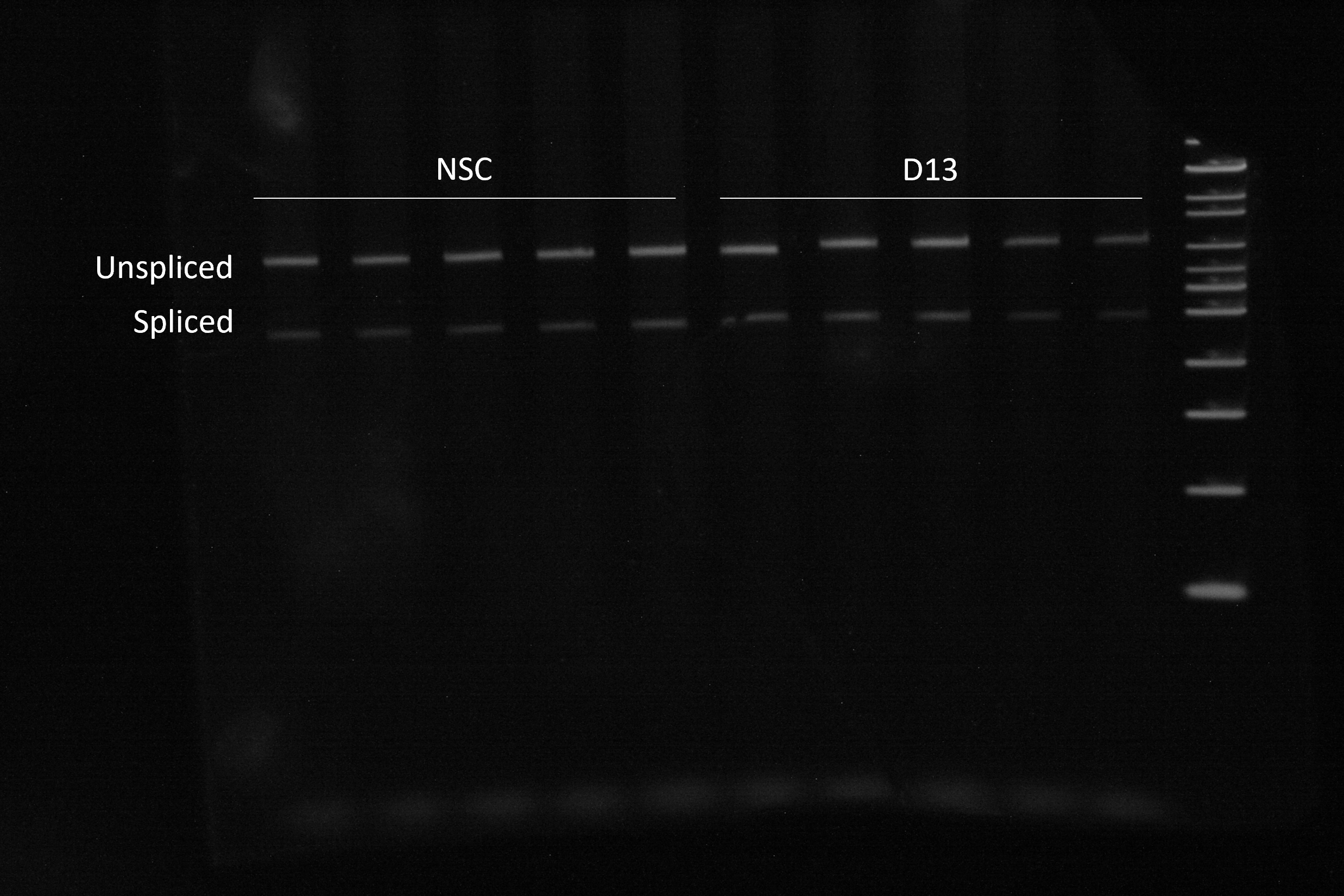

Supplement: Figure 7—figure supplement 8—source data 1. [file elife-76927-fig7-figsupp8-data1.zip › Fig 7_figure supplement 8_associated source files/Fig 7_figure supplement 8_source data 2_labeled.tif]

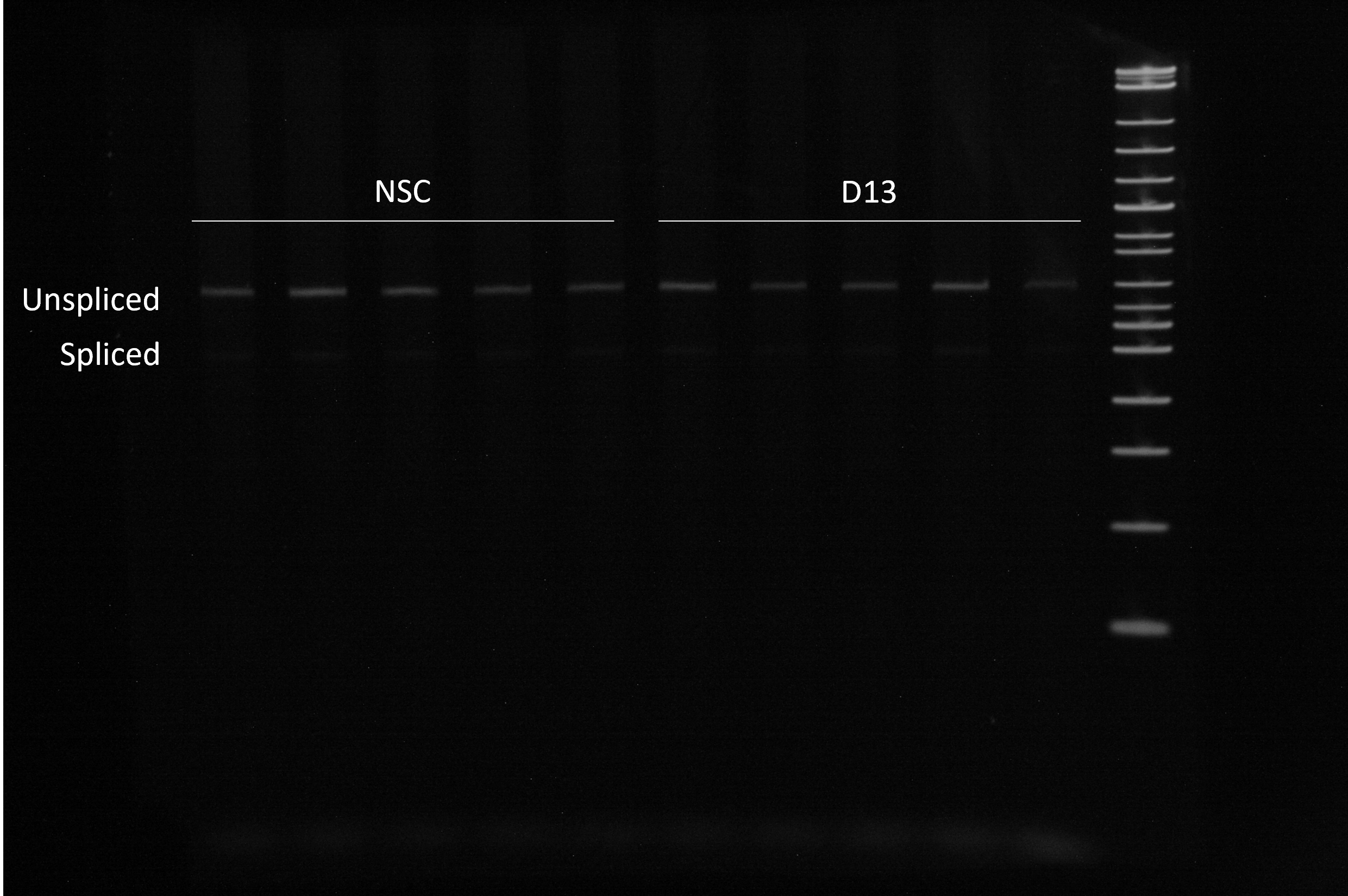

Supplement: Figure 7—figure supplement 8—source data 1. [file elife-76927-fig7-figsupp8-data1.zip › Fig 7_figure supplement 8_associated source files/Fig 7_figure supplement 8_source data 3_labeled.tif]

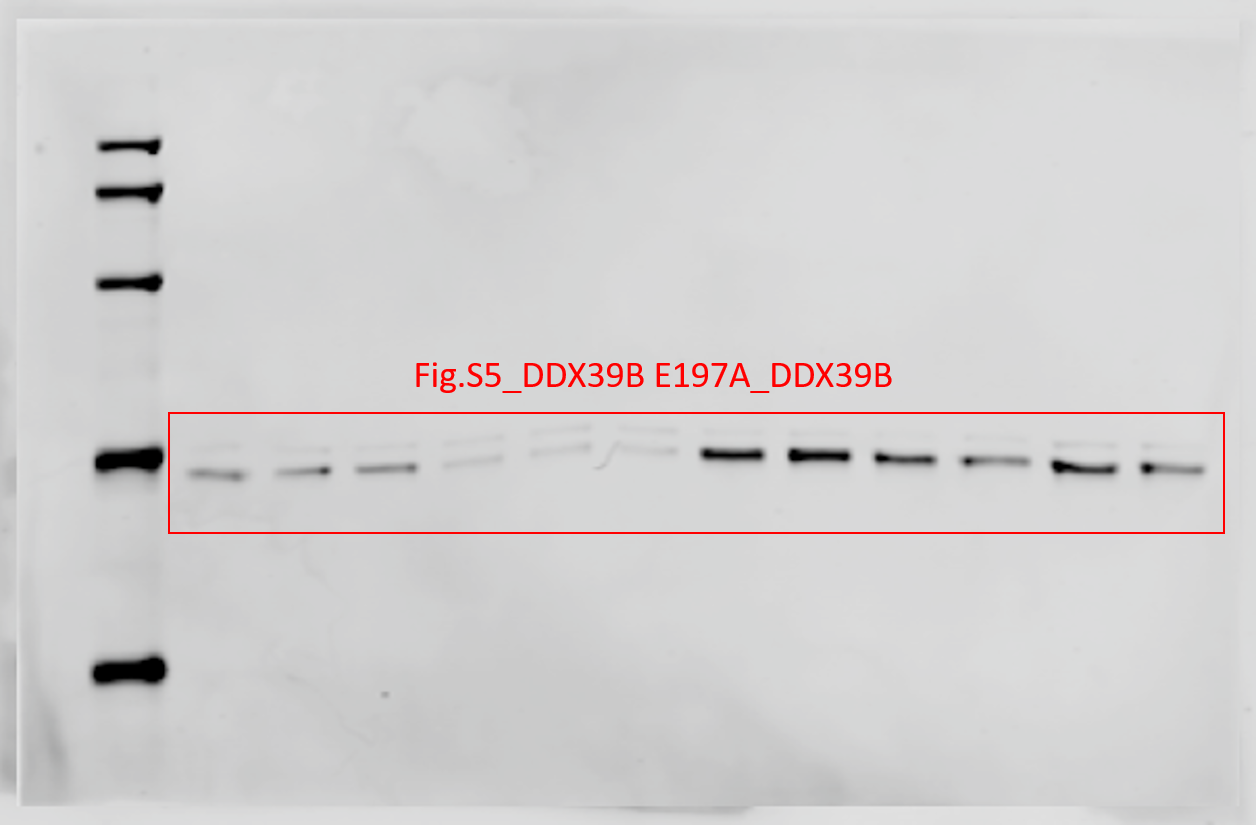

Supplement: Figure 8—figure supplement 1—source data 1. [file elife-76927-fig8-figsupp1-data1.zip › Fig 8_figure supplement 1_associated source files/Fig 8_figure supplement 1_source data 5_Labeled.tif]

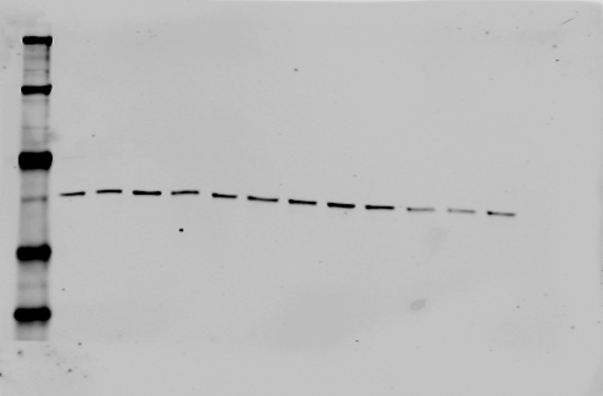

Supplement: Figure 8—figure supplement 1—source data 1. [file elife-76927-fig8-figsupp1-data1.zip › Fig 8_figure supplement 1_associated source files/Fig 8_figure supplement 1_source data 4.tif]

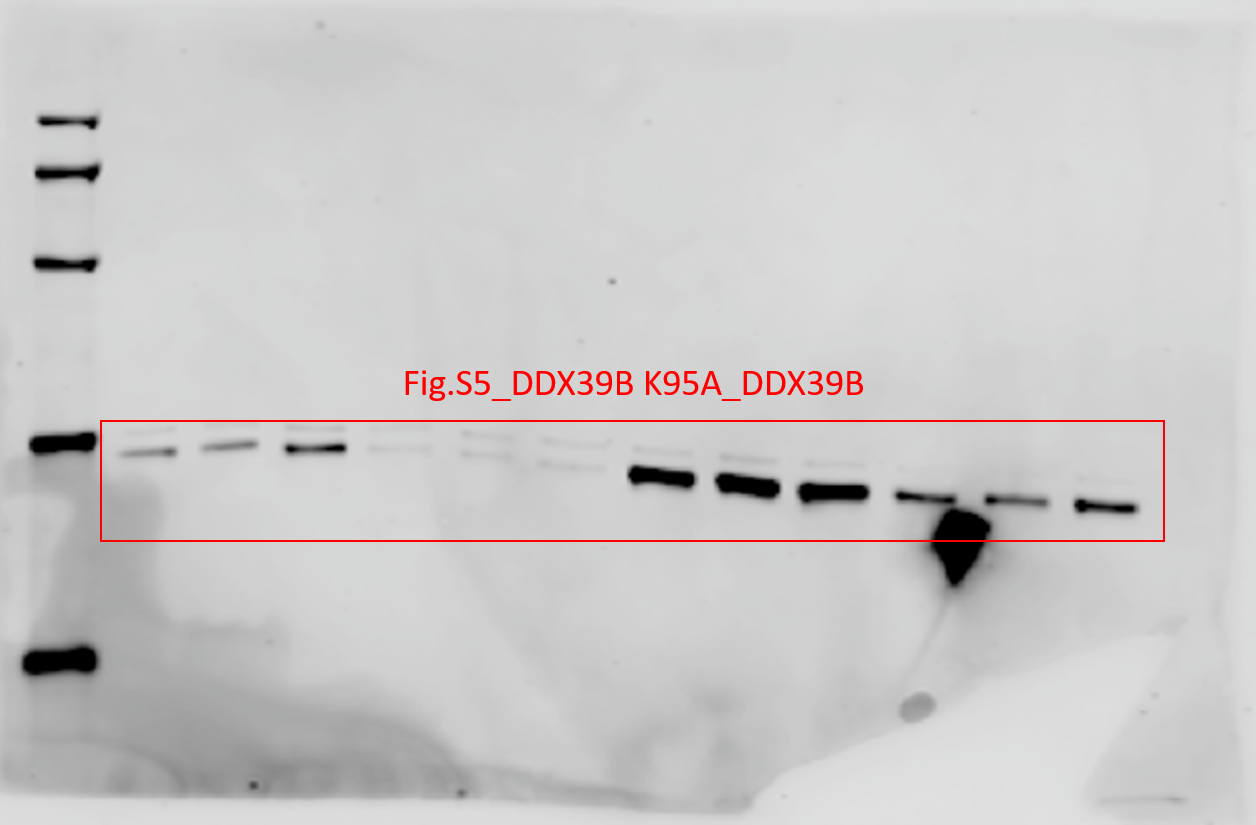

Supplement: Figure 8—figure supplement 1—source data 1. [file elife-76927-fig8-figsupp1-data1.zip › Fig 8_figure supplement 1_associated source files/Fig 8_figure supplement 1_source data 3_Labeled.tif]

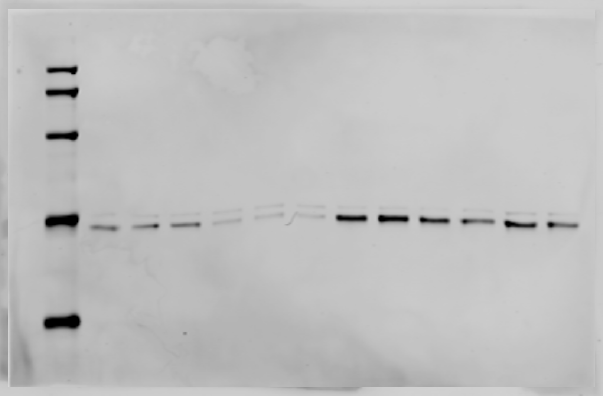

Supplement: Figure 8—figure supplement 1—source data 1. [file elife-76927-fig8-figsupp1-data1.zip › Fig 8_figure supplement 1_associated source files/Fig 8_figure supplement 1_source data 5.tif]

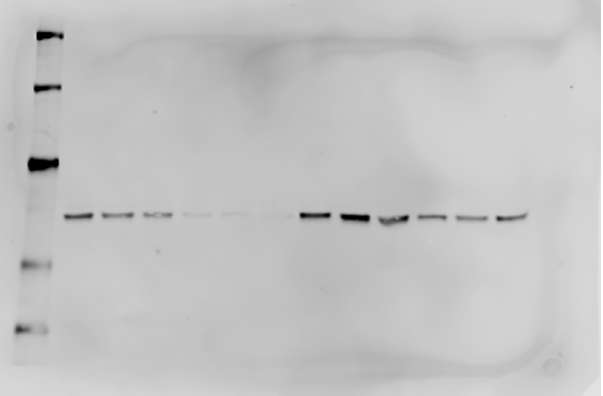

Supplement: Figure 8—figure supplement 1—source data 1. [file elife-76927-fig8-figsupp1-data1.zip › Fig 8_figure supplement 1_associated source files/Fig 8_figure supplement 1_source data 7.tif]

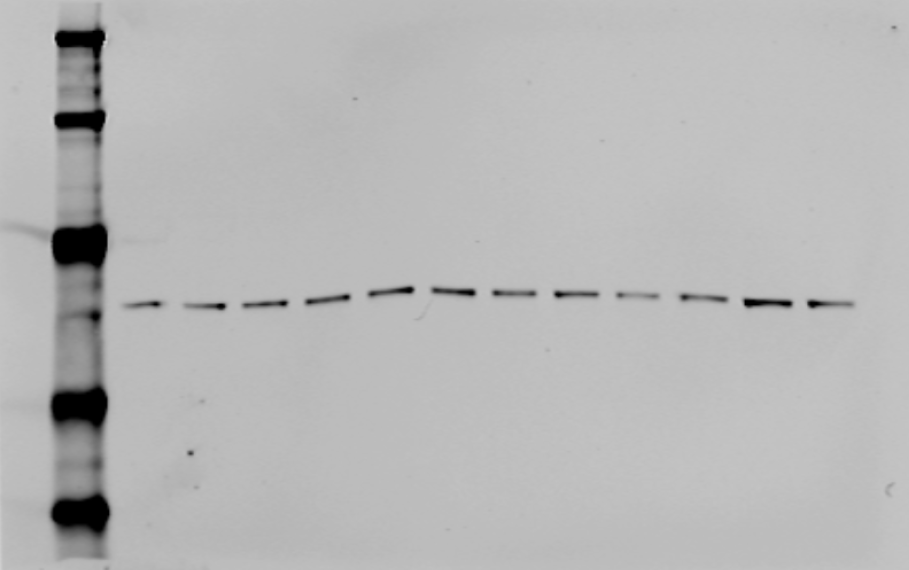

Supplement: Figure 8—figure supplement 1—source data 1. [file elife-76927-fig8-figsupp1-data1.zip › Fig 8_figure supplement 1_associated source files/Fig 8_figure supplement 1_source data 6.tif]

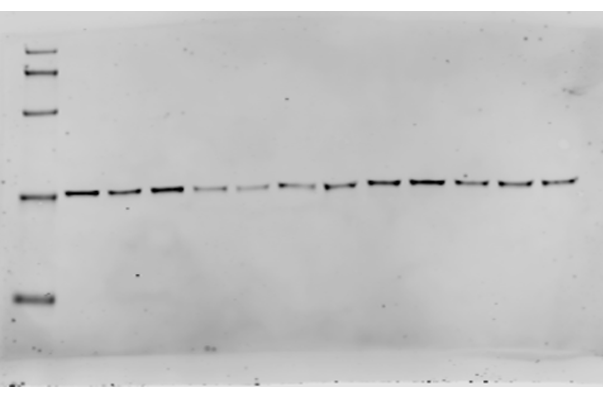

Supplement: Figure 8—figure supplement 1—source data 1. [file elife-76927-fig8-figsupp1-data1.zip › Fig 8_figure supplement 1_associated source files/Fig 8_figure supplement 1_source data 2.tif]

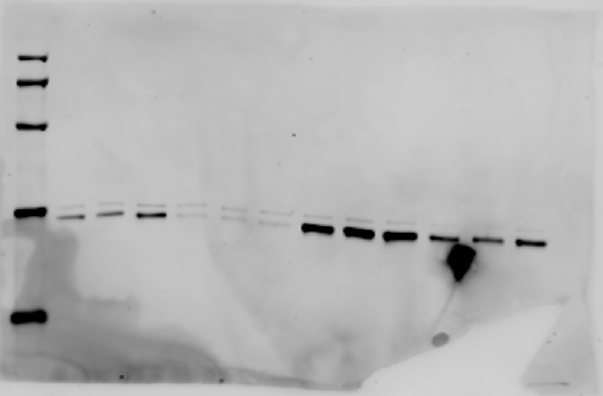

Supplement: Figure 8—figure supplement 1—source data 1. [file elife-76927-fig8-figsupp1-data1.zip › Fig 8_figure supplement 1_associated source files/Fig 8_figure supplement 1_source data 3.tif]

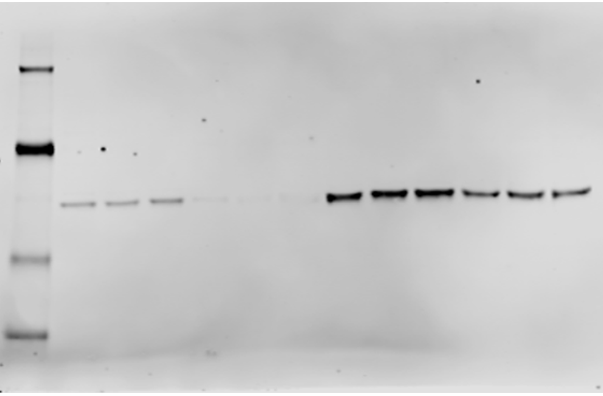

Supplement: Figure 8—figure supplement 1—source data 1. [file elife-76927-fig8-figsupp1-data1.zip › Fig 8_figure supplement 1_associated source files/Fig 8_figure supplement 1_source data 1.tif]

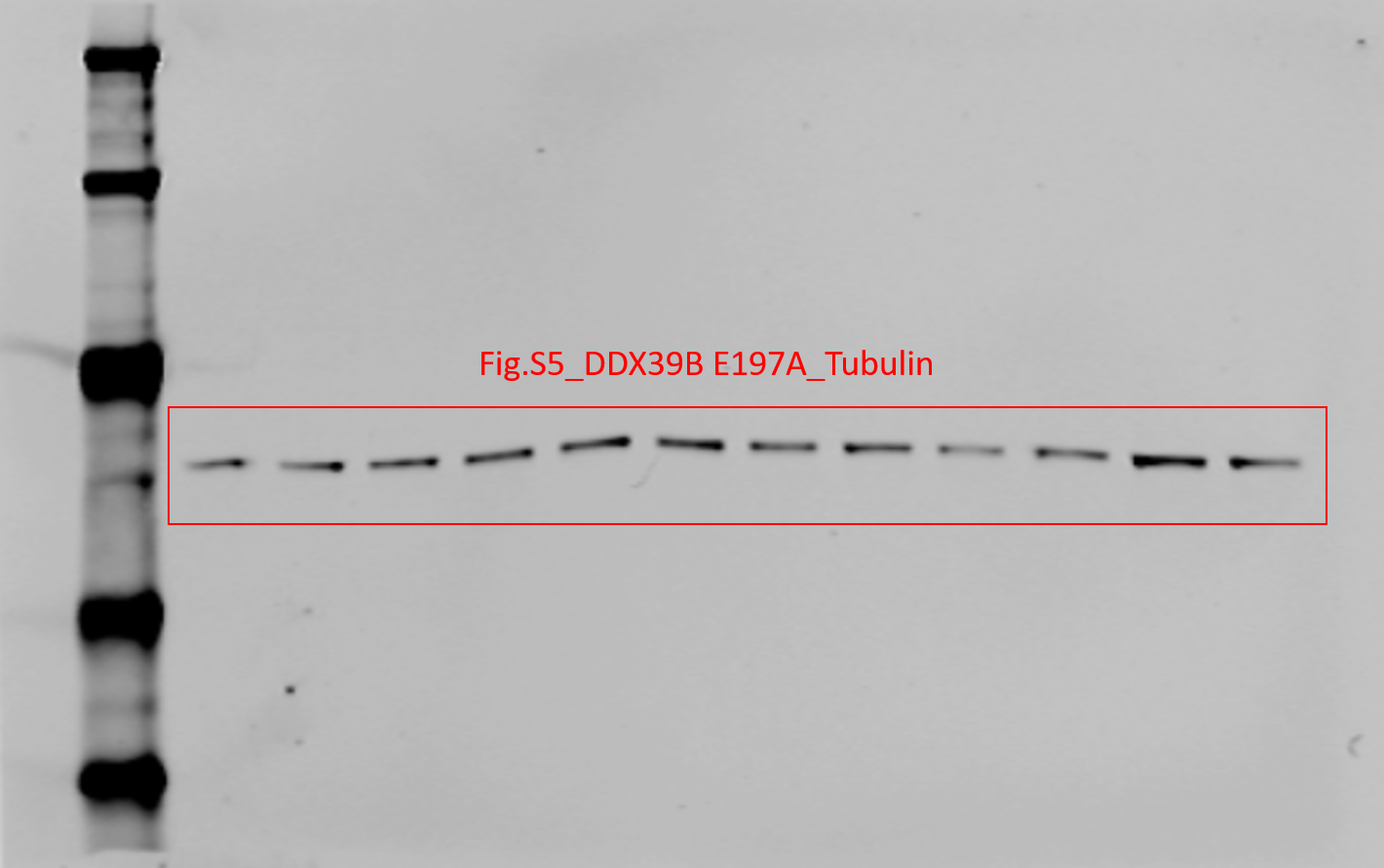

Supplement: Figure 8—figure supplement 1—source data 1. [file elife-76927-fig8-figsupp1-data1.zip › Fig 8_figure supplement 1_associated source files/Fig 8_figure supplement 1_source data 6_Labeled.tif]

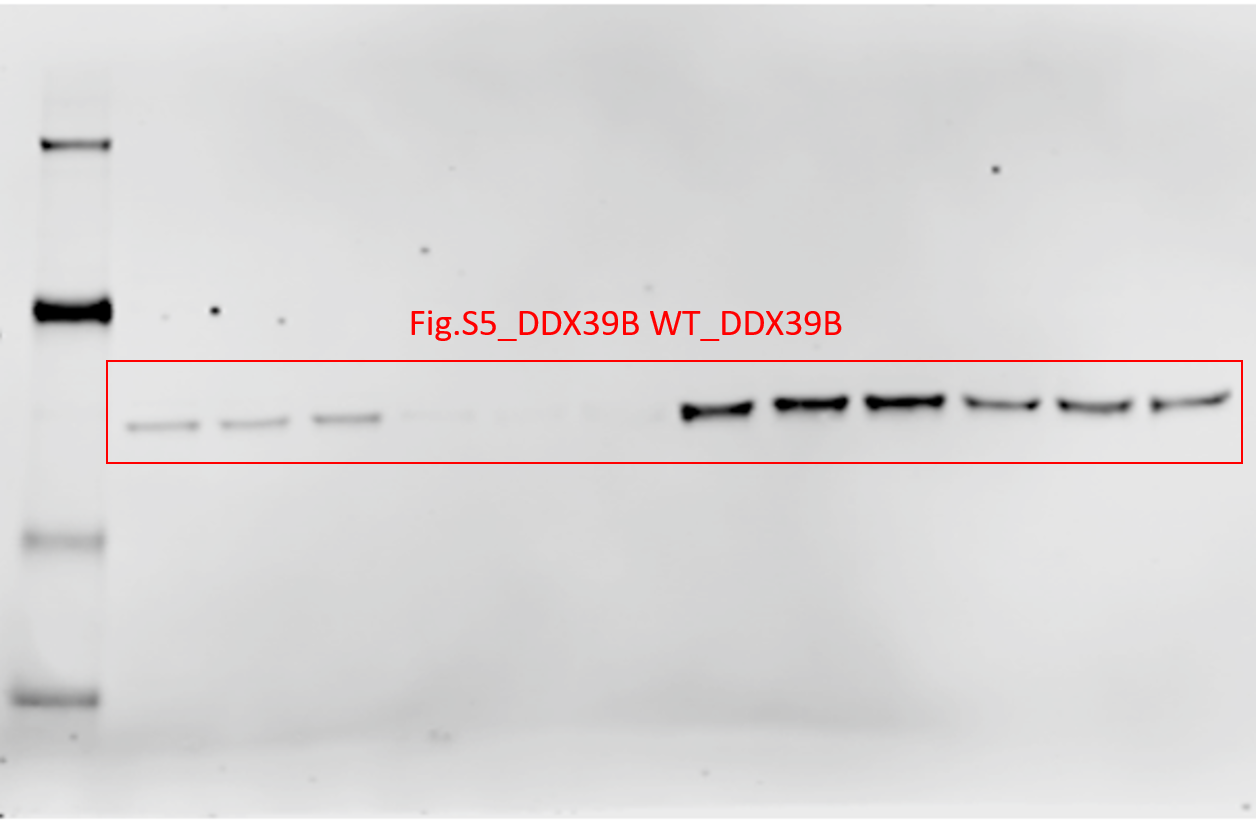

Supplement: Figure 8—figure supplement 1—source data 1. [file elife-76927-fig8-figsupp1-data1.zip › Fig 8_figure supplement 1_associated source files/Fig 8_figure supplement 1_source data 1_Labeled.tif]

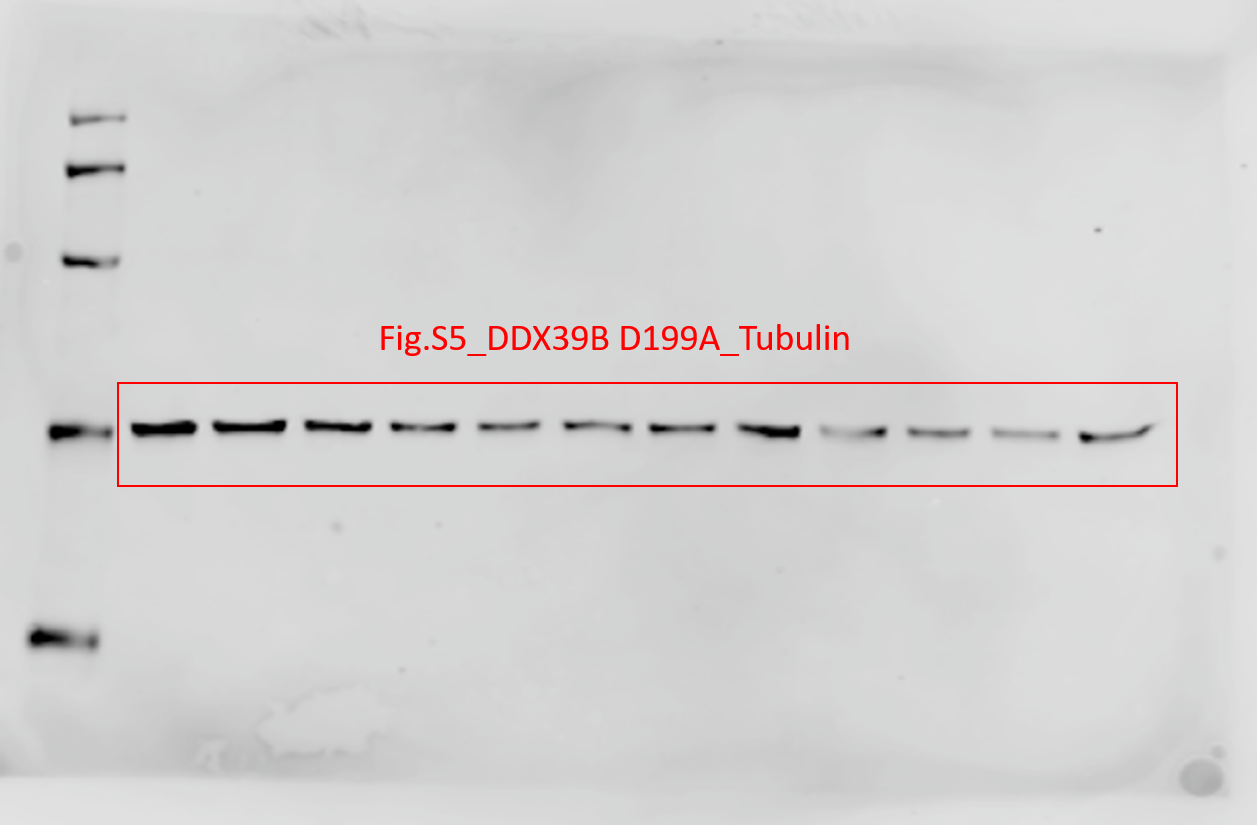

Supplement: Figure 8—figure supplement 1—source data 1. [file elife-76927-fig8-figsupp1-data1.zip › Fig 8_figure supplement 1_associated source files/Fig 8_figure supplement 1_source data 8_Labeled.tif]

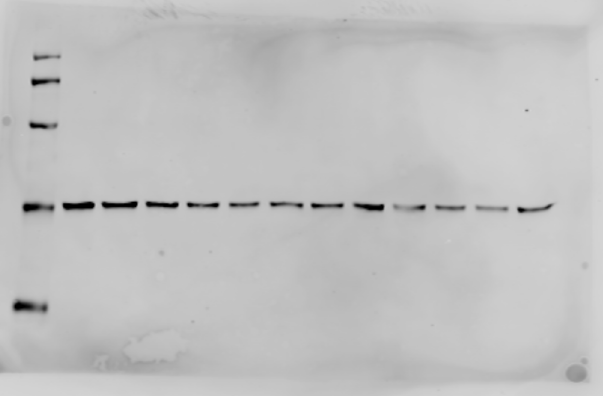

Supplement: Figure 8—figure supplement 1—source data 1. [file elife-76927-fig8-figsupp1-data1.zip › Fig 8_figure supplement 1_associated source files/Fig 8_figure supplement 1_source data 8.tif]

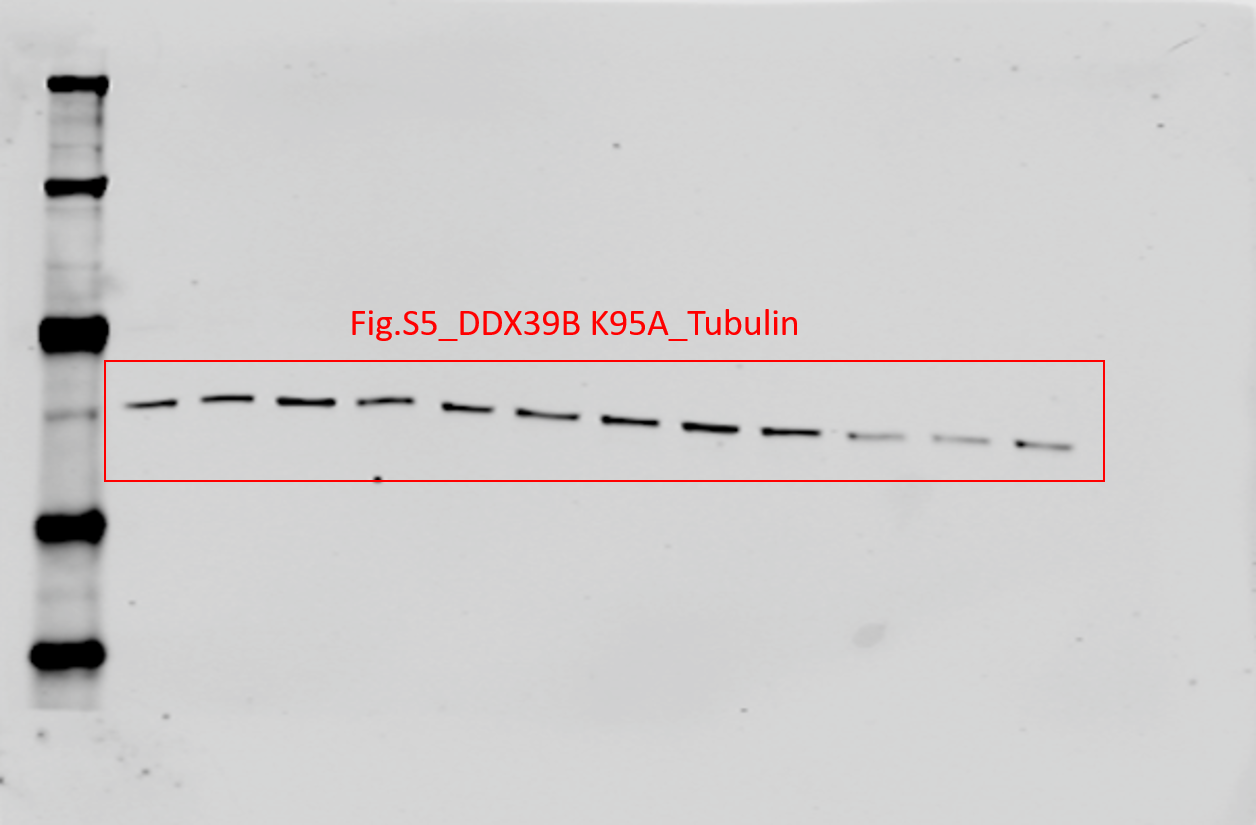

Supplement: Figure 8—figure supplement 1—source data 1. [file elife-76927-fig8-figsupp1-data1.zip › Fig 8_figure supplement 1_associated source files/Fig 8_figure supplement 1_source data 4_Labeled.tif]

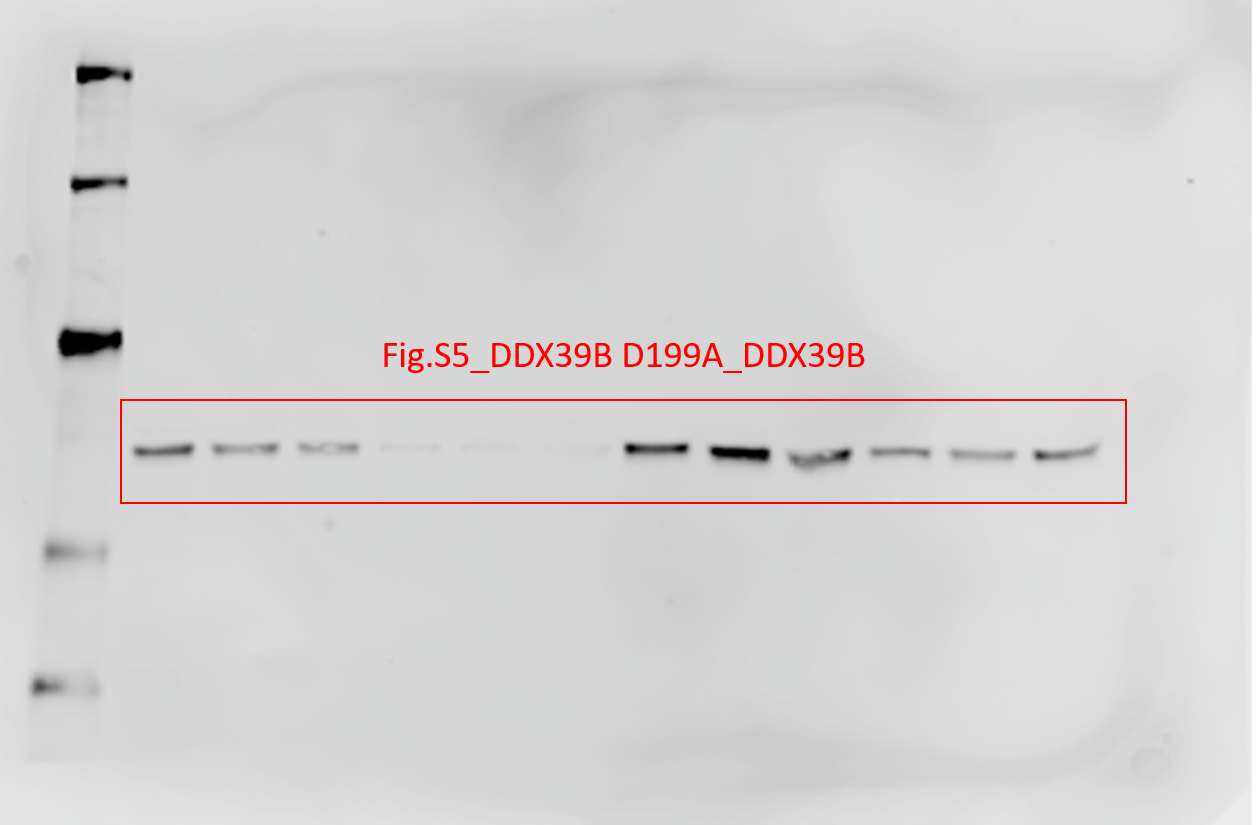

Supplement: Figure 8—figure supplement 1—source data 1. [file elife-76927-fig8-figsupp1-data1.zip › Fig 8_figure supplement 1_associated source files/Fig 8_figure supplement 1_source data 7_Labeled.tif]

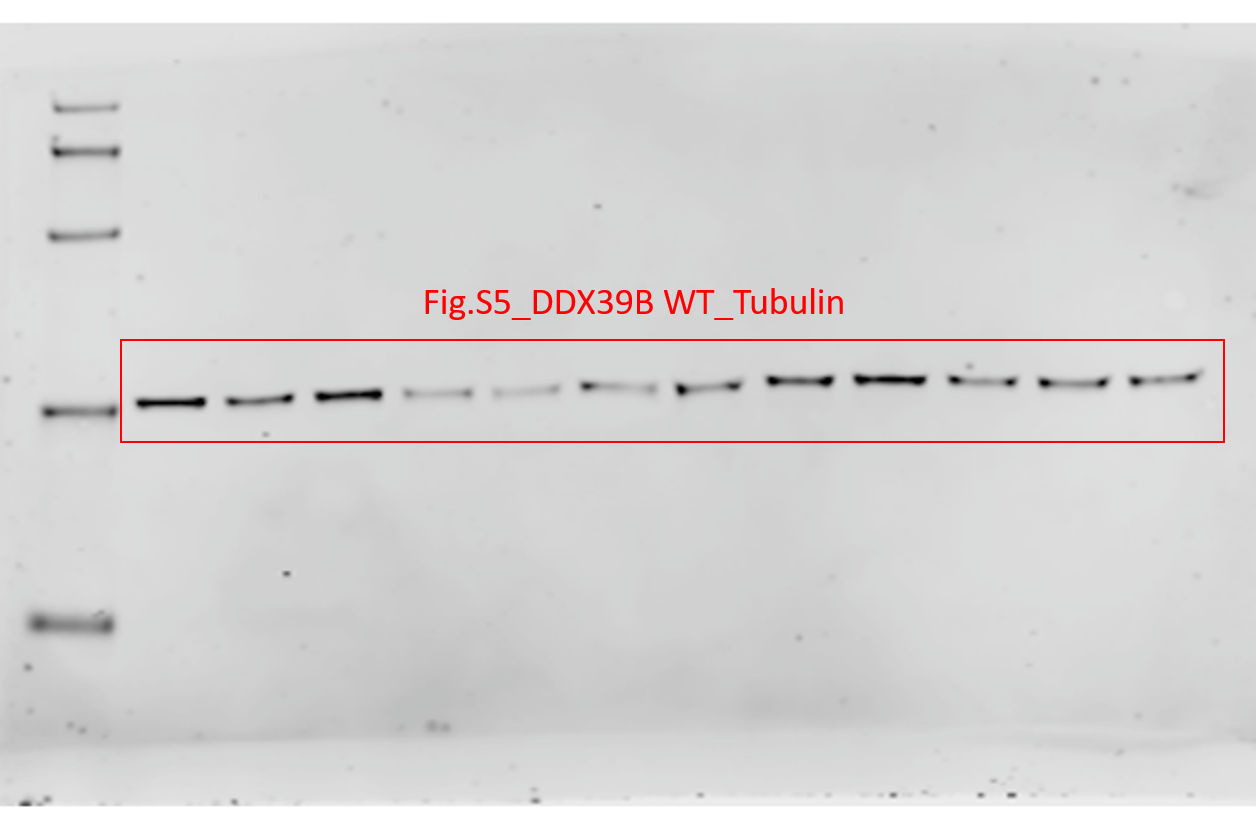

Supplement: Figure 8—figure supplement 1—source data 1. [file elife-76927-fig8-figsupp1-data1.zip › Fig 8_figure supplement 1_associated source files/Fig 8_figure supplement 1_source data 2_Labeled.tif]
