# Supplementary material for: The RNA helicase DDX39B activates FOXP3 RNA splicing to control T regulatory cell fate
Source: eLife. 2023 Jun 1;12:e76927. doi: 10.7554/eLife.76927 (PMC10234631; doi:10.7554/eLife.76927)
Supplement: Supplementary file 7. — Related to Figure 6. [file elife-76927-supp7.docx]

**Supplementary file 7. Human and Mouse FOXP3 introns. Related to Figure 6.**

**Human**

| **Intron** | **Length** | **5’ Splice site** | **5’ MaxEnt Score** | **3’ Splice site** | | **3’ MaxEnt Score** |
| --- | --- | --- | --- | --- | --- | --- |
| #1 | 6133 | AAGgugagu | 10.47 | uuccccuauugucuacgcagC | 7.85 | |
| #2 | 527 | CAGgugagg | 10.07 | ccaccugccuuucugcccagC | 7.76 | |
| #3 | 97 | CAGguaugg | 9.99 | agcccacaugugccccccagC | 4.26 | |
| #4 | 414 | CUGguaaca | 4.6 | ugcccuguccccacccacagG | 11.82 | |
| #5 | 69 | CAGgucagu | 8.94 | gacacccucuguccccccagC | 8.83 | |
| #6 | 944 | CAAgugagu | 9.1 | ggaaucuucucccuacuuagG | 6.38 | |
| #7 | 209 | CAGguaaug | 9.43 | auguuugugccauuucacagC | 8.36 | |
| #8 | 1361 | GUGgugagc | 6.64 | agccucucccccucacccagG | 6.06 | |
| #9 | 714 | CAGguaaga | 10.77 | ucauccccacccucugacagA | 6.26 | |
| #10 | 1360 | UGGguaagc | 8.84 | ccccaccccccaacuuccagG | 7.81 | |
| #11 | 180 | AAGgugagc | 9.6 | cuguccccggccuuccacagA | 8.99 | |

**Mouse**

| **Intron** | **Length** | **5’ Splice site** | **5’ MaxEnt Score** | **3’ Splice site** | **3’ MaxEnt Score** |
| --- | --- | --- | --- | --- | --- |
| #1 | 5965 | CAGguaaag | 11.08 | ucucaucuccuccauccaagC | 4.76 |
| #2 | 439 | CAGguuggu | 8.08 | uuccacuguuaucccugcagC | 9.58 |
| #3 | 512 | CAGgugagg | 10.07 | caucucccuucucucuccagC | 11.96 |
| #4 | 96 | CAGguaugg | 9.99 | augguccauguguuccccagC | 6.62 |
| #5 | 373 | CUGguaaca | 4.6 | uacccugucucuacccacagG | 12.09 |
| #6 | 98 | CAGgugagu | 10.67 | ccauauucucccauccccagC | 6.9 |
| #7 | 790 | CAAgugagu | 9.1 | ggaaucuucuccuuacucagG | 5.05 |
| #8 | 140 | CAGguaaug | 9.43 | uuugguguacauccccacagC | 7.81 |
| #9 | 2196 | GUGgugagu | 8.95 | caccccucccaaccccucagG | 8 |
| #10 | 632 | CAGgucagu | 8.94 | ucuaugccaaccuucugcagA | 6.6 |
| #11 | 414 | UGGguaagc | 8.84 | caccugacuccaacuuccagG | 7.1 |
| #12 | 148 | AAGgugagu | 10.47 | ucccggucccccuuccacagA | 9.92 |
